# The novel rexinoid MSU-42011 is effective for the treatment of preclinical Kras-driven lung cancer

**DOI:** 10.1038/s41598-020-79260-8

**Published:** 2020-12-17

**Authors:** Jessica A. Moerland, Di Zhang, Lyndsey A. Reich, Sarah Carapellucci, Beth Lockwood, Ana S. Leal, Teresa Krieger-Burke, Bilal Aleiwi, Edmund Ellsworth, Karen T. Liby

**Affiliations:** 1 Department of Pharmacology and Toxicology, Michigan State University, B430 Life Science Building, 1355 Bogue Street, East Lansing, MI 48824 USA; 2 In Vivo Facility, Michigan State University, East Lansing, MI USA; 3 Medicial Chemistry Core, Michigan State University, East Lansing, MI USA

**Keywords:** Cancer, Drug discovery

## Abstract

Effective drugs are needed for lung cancer, as this disease remains the leading cause of cancer-related deaths. Rexinoids are promising drug candidates for cancer therapy because of their ability to modulate genes involved in inflammation, cell proliferation or differentiation, and apoptosis through activation of the retinoid X receptor (RXR). The only currently FDA-approved rexinoid, bexarotene, is ineffective as a single agent for treating epithelial cancers and induces hypertriglyceridemia. Here, we used a previously validated screening paradigm to evaluate 23 novel rexinoids for biomarkers related to efficacy and safety. These biomarkers include suppression of inducible nitric oxide synthase (iNOS) and induction of sterol regulatory element-binding protein (SREBP). Because of its potent iNOS suppression, low SREBP induction, and activation of RXR, MSU-42011 was selected as our lead compound. We next used MSU-42011 to treat established tumors in a clinically relevant *Kras*-driven mouse model of lung cancer. *KRAS* is one of the most common driver mutations in human lung cancer and correlates with aggressive disease progression and poor patient prognosis. Ultrasound imaging was used to detect and monitor tumor development and growth over time in the lungs of the A/J mice. MSU-42011 markedly decreased the tumor number, size, and histopathology of lung tumors compared to the control and bexarotene groups. Histological sections of lung tumors in mice treated with MSU-42011 exhibited reduced cell density and fewer actively proliferating cells compared to the control and bexarotene-treated tumors. Although bexarotene significantly (*p* < 0.01) elevated plasma triglycerides and cholesterol, treatment with MSU-42011 did not increase these biomarkers, demonstrating a more favorable toxicity profile in vivo. The combination of MSU-42011 and carboplatin and paclitaxel reduced macrophages in the lung and increased activation markers of CD8+T cells compared to the control groups. Our results validate our screening paradigm for in vitro testing of novel rexinoids and demonstrate the potential for MSU-42011 to be developed for the treatment of KRAS-driven lung cancer.

## Introduction

Lung cancer is the leading cause of cancer-related deaths worldwide, with over 228,000 new cases diagnosed and more than 135,000 deaths per year in the United States alone^1^. Cytotoxic chemotherapy and a limited number of targeted agents have been the standard of care treatment for non-small-cell lung cancer (NSCLC)^2–4^. However, chemotherapy is poorly tolerated by patients and has limited efficacy^4^. In 2014, immune checkpoint inhibitors were first approved and are now used alone or in combination with chemotherapy to treat lung cancer^5^. At best, only 20% of patients respond to immunotherapy^6,7^, so there is an urgent need to develop new therapies.

Rexinoids are selective ligands for the retinoid X receptor (RXR), a member of the nuclear receptor family. RXR homodimerizes or heterodimerizes with other nuclear receptors, and upon ligand binding, the dimers act as transcription factors, regulating genes involved in inflammation, cell proliferation or differentiation, and apoptosis^8^. This profile makes RXR an attractive target for anti-cancer therapies^9^. The rexinoid bexarotene is FDA-approved for the treatment of cutaneous T-cell lymphoma^10^. When tested in clinical trials for the treatment of lung cancer in combination with standard of care chemotherapies^11–16^, bexarotene significantly increased overall survival in the 30–40% of patients^13,14^ who developed hypertriglyceridemia. The combination of bexarotene and erlotinib, a tyrosine kinase inhibitor which targets the epidermal growth factor receptor, increased overall survival by 583–1460 days in a subset of heavily pretreated patients with advanced lung cancer^15^. These results are important, as increases in overall survival are frequently measured in weeks or a few months for newly approved therapeutics for lung cancer.

Although bexarotene was effective in subsets of patients in multiple clinical trials, this drug was not approved for treatment of lung cancer because of a lack of survival benefit in the majority of patients. The rexinoids LG100268 and IRX194204 are more potent than bexarotene and have single-agent activity in preclinical models of lung cancer^17^ and breast cancer^18,19^. However, elevated triglyceride levels, lack of toxicity studies, and intellectual property issues have prevented the clinical development of these more potent rexinoids^9,20^.

To overcome these challenges, we have synthesized new rexinoids and optimized a set of in vitro screening assays that correlate with efficacy and toxicity in vivo^21^. This screening system quantifies inhibition of the inflammatory mediator inducible nitric oxide synthase (iNOS) and induction of sterol regulatory element binding protein (SREBP), involved in triglyceride synthesis. Inflammation plays an important role in carcinogenesis^22–24^ and creates a favorable microenvironment in which tumors can thrive^25^. The ability of rexinoids to inhibit lipopolysaccharide-induced production of nitric oxide (NO) in RAW264.7 macrophage-like cells directly correlates with anti-cancer activity in the clinically relevant A/J mouse model of lung cancer^21^. SREBP is a transcription factor that regulates cellular lipid metabolism and homeostasis^26^ and therefore is a surrogatge in vitro biomarker for predicting hypertriglyceridemia.

Activating mutations in the *KRAS* gene can be found in 35% of all lung cancers and in up to 93% of NSCLC cases in smokers^27,28^ These mutations drive tumor development and disease progression^29–32^, as meta-analysis in patients with NSCLC has shown that *KRAS* mutations are associated with poor prognosis and worse overall survival compared to those without *KRAS* mutations^33,34^. Attempts to directly target *KRAS* in NSCLC with a small molecule have been unsuccessful historically, in part because of the lack of a pocket on KRAS to which small molecules can bind^35,36^. The development of pharmacological inhibitors that specifically target mutant KRAS proteins^37,38^ has justifiably renewed interest in this field. However, challenges remain as the *KRAS*^*G12C*^ mutations targeted by current inhibitors represent approximately 45%, but not all *KRAS* mutations in NSCLC^38,39^, and the likely development of resistance to these novel inhibitors^40^ must be addressed.

In our studies, we synthesized and screened 23 new rexinoids for their ability to suppress iNOS production and induce SREBP expression in vitro. A subset of compounds was then tested for RXRα activity. MSU-42011 was selected as the lead compound and tested in vivo in the A/J mouse model of NSCLC. These mice develop *Kras* mutations and subsequent lung adenocarcinomas after being challenged with the carcinogen vinyl carbamate^41^. After initiation, tumor growth in the lungs was monitored by ultrasound imaging. When used to treat established lung tumors, MSU-42011 alone significantly decreased overall tumor burden and altered immune cell populations in the lung when used in combination with chemotherapy.

## Results

### Rexinoids upregulate the expression of SREBP in liver cells by activating RXR

**Figure 1:**
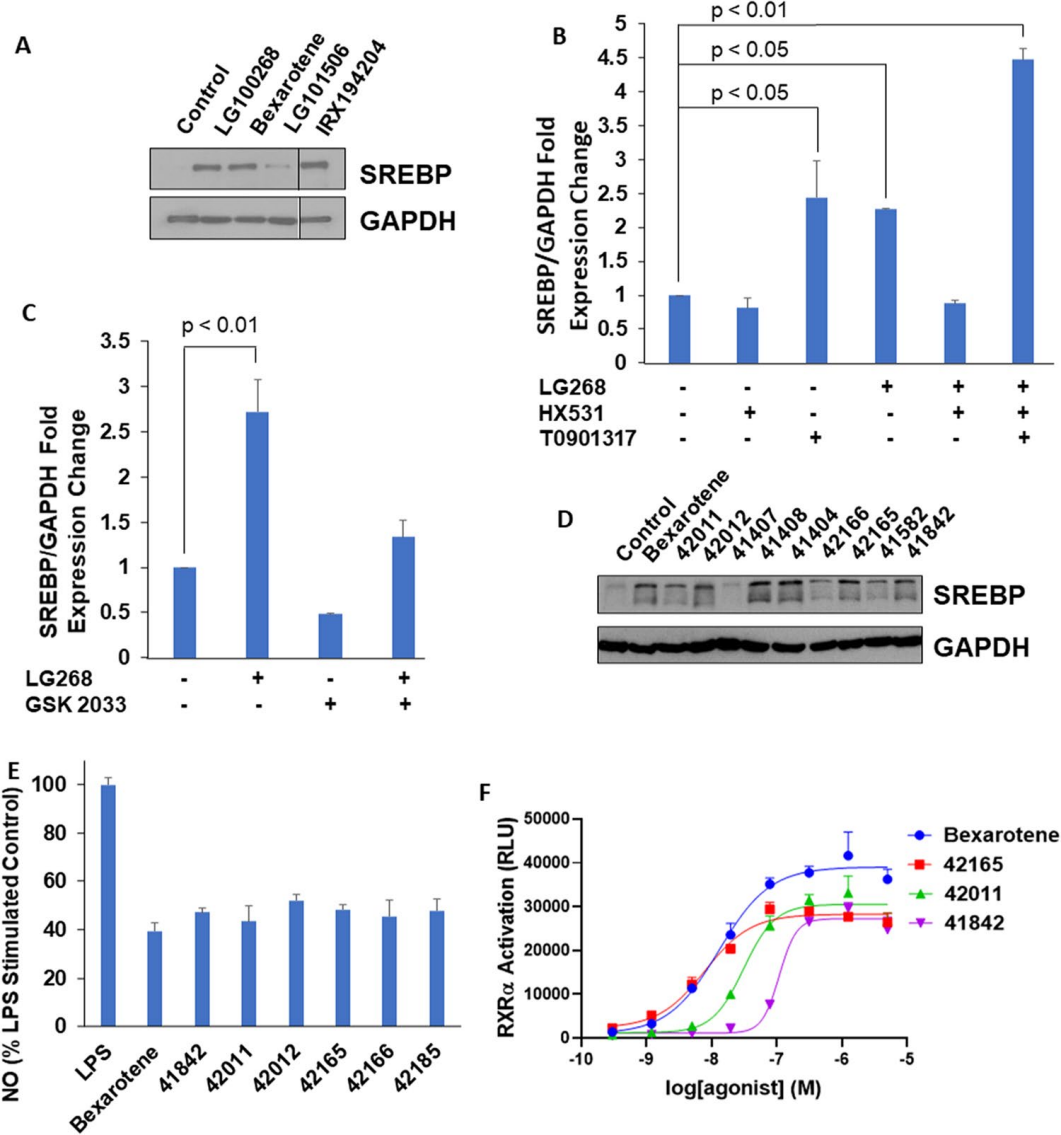
In vitro screening assays. (**A**): Established rexinoids alter SREBP protein expression. HepG2 cells were treated with 300 nM rexinoids for 24 hr and SREBP mRNA expression was quantified using RT-PCR. (**B**) Differences in SREBP protein expression induced by novel rexinoids. HepG2 cells were treated with 300 nM rexinoids for 24 hr and SREBP/GAPDH protein expression was measured by western blot. (**C**) SREBP mRNA expression. HepG2 cells were treated with 300 nM LG100268 (an RXR agonist) and/or 500 nM GSK 2033 (an LXR antagonist). SREBP mRNA expression was quantified using RT-PCR. (**D**) SREBP mRNA expression. HepG2cells were treated with 300 nM LG100268 (an RXR agonist), 100 nM HX 531 (an RXR antagonist), and/or 500 nM T0901317 (an LXR agonist). (**E**) iNOS inhibition of select novel compounds. RAW264.7 macrophage-like cells were treated with 300 nM rexinoids and stimulated with LPS for 24 h. NO in the media of treated cells was measured by the Griess assay. (**F**) RXR activation of select novel compounds. Reporter cells were treated with 0–5000 nM rexinoids and RXRα activation was measured using a commercially available kit.

Although we have confirmed that the iNOS assay correlates with in vivo efficacy^21^, few investigators have used the induction of SREBP in vitro as a biomarker to predict the ability of novel rexinoids to increase triglycerides in vivo. Elevated triglycerides are a side-effect found with many RXR agonists in preclinical models and by bexarotene in humans^13,14^. In order to validate the utility of SREBP as an in vitro biomarker for increased triglycerides in vivo, established rexinoids known to induce hypertriglyceridemia (bexarotene, LG100268, and IRX194204) and LG101506 (a rexinoid that does not induce hypertriglyceridemia in vivo)^21,42^ were directly compared to evaluate their ability to induce SREBP protein in vitro (Fig. 1A). HepG2 human liver cells were used, as the liver is the major organ that regulates lipid homeostasis. When HepG2 cells were treated with 300 nM rexinoids for 24 h, bexarotene, LG100268, and IRX194204 all increased SREBP protein levels compared to cells treated with the DMSO control while LG101506 did not (Fig. 1A). These data are consistent with the known ability of these compounds to elevate triglycerides in vivo^21,42^, as shown in Supplemental Fig. 1.

The mechanism by which rexinoids induce hypertriglyceridemia is not fully known, but ligand-induced heterodimerization of RXR with the liver X receptor (LXR) may play an important role, as SREBP is a known target of LXR^26^. To test this hypothesis, we treated HepG2 cells with an LXR agonist (T0901317, 500 nM), an RXR antagonist (HX 531, 100 nM), or an LXR antagonist (GSK 2033, 500 nM), either alone or in combination with the rexinoid LG100268 (300 nM). After 8 h of treatment, SREBP mRNA was measured by RT-PCR (Fig. 1B). As expected, LG100268 significantly (*p* < 0.01) increased SREBP expression at the transcriptional level by 2.5 fold compared to the vehicle control. The LXR agonist T0901317 also induced a 2.5 fold increase in SREBP mRNA (*p* < 0.05), and combining T0901317 with LG100268 induced SREBP mRNA expression by ~ 4.5 fold (*p* < 0.01). The RXR antagonist HX 531 alone did not alter SREBP mRNA expression and reversed the induction of SREBP by LG100268. Interestingly, the LXR antagonist GSK 2033 alone lowered SREBP mRNA expression by ~ 50%, and the combination of GSK 2033 with LG100268 partially reversed the induction of SREBP by LG100268 (Fig. 1C). Similar trends are observed with recently synthesized next-generation rexinoids which also elevate SREBP expression in vitro.

### Synthesis and in vitro evaluation of novel rexinoids

To develop more effective rexinoids with fewer side effects, 23 novel analogs were synthesized (Table 1) using fragments previously used with known RXR agonists. The goals of our structure–activity relationship (SAR) studies were to explore and discover new scaffolds that not only bind to RXR but also are able to independently modulate both iNOS and SREBP activity in accordance with a previously described screening paradigm^21^. To characterize the effect of the novel rexinoids on SREBP elevation in vitro, HepG2 cells were treated with 300 nM rexinoids for 8 h and SREBP mRNA levels were measured by RT-PCR. SREBP expression ranged from no change to a 4.6-fold induction compared to the vehicle control when treated with the novel compounds. These results were confirmed by western blot for several compounds that showed differences in SREBP expression despite minor structural modifications (Fig. 1D). Analogs MSU- 41402, 41582, 41407, 41566, 41844, 41845, 42011, and 42166 induced an SREPB mRNA fold expression change of 1–1.5 normalized to a vehicle control, while bexarotene induced an mRNA fold expression change of > 2 (Table 1). The low SREBP fold-change of HepG2 cells treated with this subset of compounds compared to bexarotene suggests that these select analogs will not induce hypertriglyceridemia compared to bexarotene in vivo^21^, which we will confirm in vivo.

**Table 1:** Activity of new rexinoids in iNOS, RXR and SREBP in vitro assays.

Rexinoid	Structure	MW	Efficacy	Safety
			iNOS suppression IC_50_ (nM)	SREBP activation Fold induction vs. Control
Bexarotene		348.5	143 ± 19	2.23 ± 0.5
LG100268		363.5	83 ± 14	3.06 ± 0.7
MSU-41404		320.5	340 ± 25	3.07 ± 0.5
MSU-41566		336.2	239 ± 53	1.12 ± 0.5
MSU-41405		338.4	267 ± 32	1.87 ± 0.3
MSU-41407		338.4	334 ± 17	1.08 ± 0.1
MSU-41406		352.5	252 ± 55	1.67 ± 0.2
MSU-41408		324.4	300 ± 79	2.55 ± 0.7
MSU-41403		381	331 ± 45	2.68 ± 0.2
MSU-41845		369.2	338 ± 31	1.45 ± 0.2
MSU-41583		310	290 ± 61	1.59 ± 0.5
MSU-41567		418.5	271 ± 92	1.58 ± 0.2
MSU-41402		353.5	343 ± 62	1.37 ± 0.3
MSU-41564		354.5	248 ± 60	2.62 ± 0.7
MSU-42185		369.2	128 ± 49	1.65 ± 0.1
MSU-41842		368.2	161 ± 42	2.15 ± 0.7
MSU-41843		380.2	274 ± 33	2.48 ± 1.8
MSU-42012		382.3	141 ± 27	4.6 ± 2.3
MSU-42011		382.5	158 ± 35	1.49 ± 0.6
MSU-41582		368.2	295 ± 54	1.38 ± 0.4
MSU-42165		383.3	172 ± 13	2.46 ± 0.5
MSU-42166		381.2	221 ± 51	1.46 ± 0.2
MSU-41844		378.1	389 ± 53	0.96 ± 0.5
MSU-41565		397.6	366 ± 49	1.59 ± 0.2
MSU-41846		383.2	347 ± 31	1.63 ± 0.3

To measure iNOS suppression, RAW264.7 macrophage-like cells were treated with twofold serial dilutions (0–1000 nM) of rexinoids and stimulated with LPS (1 ng/ml) for 24 h. NO in the media was measured by the Griess assay. IC50 values were calculated, and the average IC50 values (mean ± SE) of three independent experiments are shown. To measure SREBP induction, HepG2 liver cancer cells were treated with 300 nM rexinoids for 8 h, and SREBP mRNA expression was determined by RT-PCR. Values represent mean ± SD of 3 independent experiments.

To evaluate the anti-inflammatory effects of the novel rexinoids, we utilized the iNOS suppression assay. Inhibition of iNOS in vitro directly correlates with efficacy in our preclinical model of Kras-driven lung cancer^21^. RAW264.7 macrophage-like cells were treated with 0–1 μM rexinoids and stimulated with 1 ng/mL lipopolysaccharide for 24 h, and then NO was measured in the media. Of the 23 compounds tested, analogs MSU- 41842, 42011, 42012, 42165, 42166, and 42185 were the most potent, with IC_50_ values ranging from 80–225 nM (Table 1). These specific compounds were then directly tested at a single concentration in the iNOS suppression assay and demonstrated similar efficacy at 300 nM when compared to bexarotene (Fig. 1E).

These studies summarized in Table 1 reveal, by inspection, the following: (1) the decalin ring system of bexarotene and LG100268 can be, as others have reported^43,44^, replaced with alternate scaffolds exhibiting excellent activity in the iNOS assay; (2) the relationship between iNOS and SREBP activity can be separated, even from analogs of higher iNOS activity (see for example bexarotene vs. MSU-41566 and MSU-42011); (3) the core vinyl of bexarotene or cyclopropyl of LG100268, each acting as a bridge between the aromatic ring of each can be replaced with a nitrogen^45^ resulting in derivatives with good iNOS activity (MSU-42185, -41842, -42012, -42011); (4) substitution on nitrogen with different alkyl substituents, in combination with alternate aromatics on each position of the nitrogen bridge, allows for independent modulation of both iNOS and SREBP activity. More specifically, we find that as the alkyl substitution on the nitrogen bridge was changed in the series from ethyl (MSU-41564), isopropyl (MSU-42185), *n*-propyl (MSU-41842**)**, isobutyl (MSU-42011**)** and then *n*-butyl (MSU-42012), the highest iNOS activity and greatest selectivity versus SREBP is observed by substitution with isopropyl, *n*-propyl and isobutyl when substituted with a pyridine on the nitrogen bridge. Although a similar trend of iNOS activity may exist in the pyrimidine-substituted series (MSU-41582, -42165, -42166), more systematic studies are required to verify. In all, the nitrogen-branched series allows for three points of diversification to more fully study and fine-tune the separation between SREBP and iNOS activity and SAR understanding of these activities.

Select novel compounds that demonstrated low SREBP induction and robust iNOS suppression were then tested for their ability to activate RXRα (Fig. 1F). Stably transfected reporter cells were treated with 0–5 µM rexinoids, and RXRα activation was measured. The new compounds with the highest RXRα activation were analogs MSU- 41842, 42011, and 42165, with EC_50_ values ranging from 10–110 nM. Bexarotene was included as a positive control.

Based on the data from the in vitro screening assays, the novel rexinoid MSU-42011 was selected for testing in vivo because of its potent iNOS suppression, low SREBP induction, and activation of RXRα. Before starting efficacy studies, the oral exposure of MSU-42011 was measured in vivo*.* Mice were fed diet containing MSU-42011 (100 mg/kg of diet or ~ 25 mg/kg body weight) for 4 days. Plasma and lung homogenates were analyzed by liquid chromatography-mass spectrometry. Adjusted steady state concentrations of MSU-42011 were 78 nM in the plasma and 72 nM in the lung, which are similar to values observed with other rexinoids that are effective in a preclinical model of lung cancer^17^.

### Ultrasound imaging used to monitor lung tumor onset and development in A/J mice

**Figure 2:**
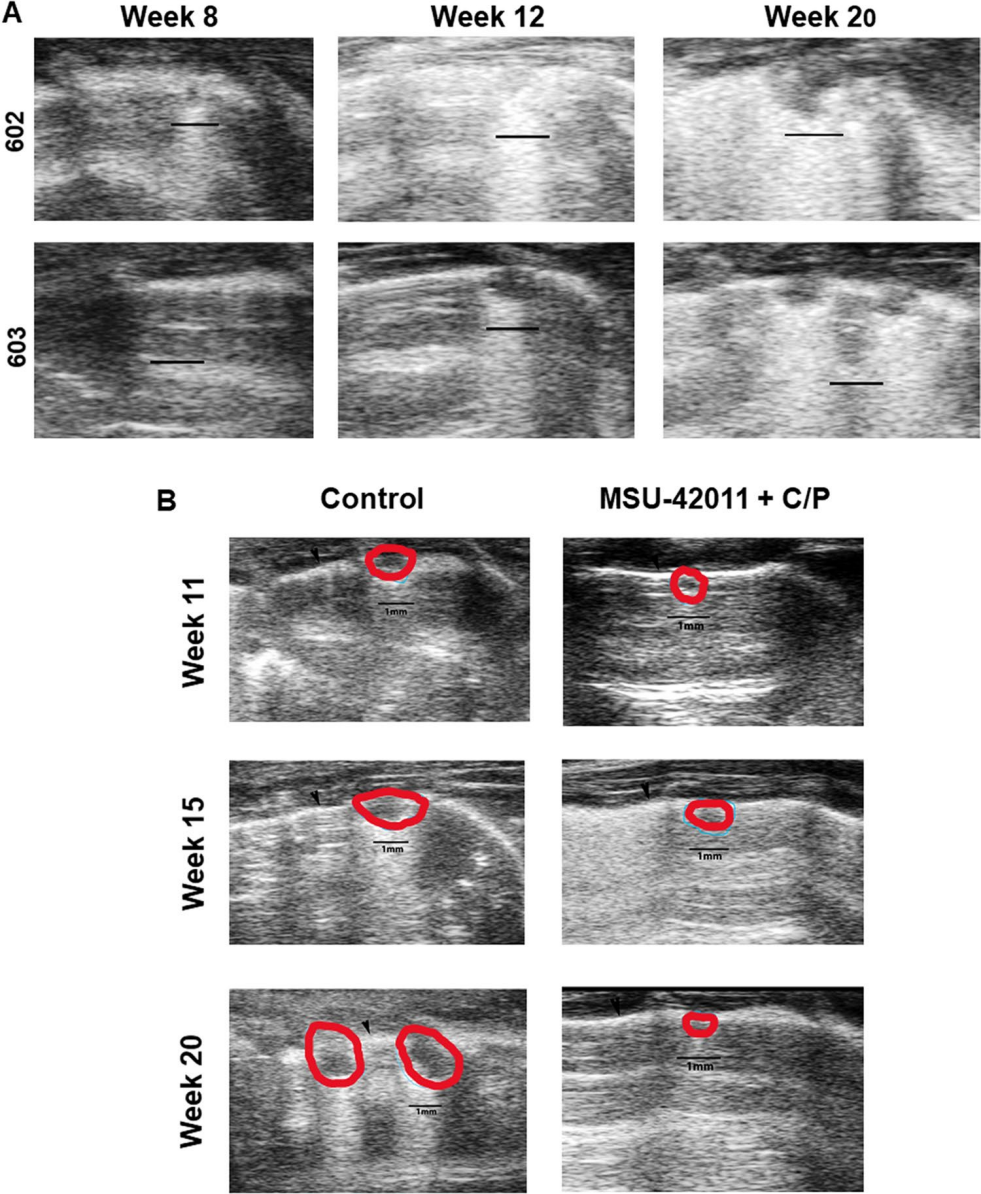
Ultrasound shows efficacy of MSU-42011 for treatment of established Kras-driven lung tumors. A/J mice were injected with vinyl carbamate to initiate the development of lung tumors. Ultrasound was used to monitor the progression of lung tumors 8, 12 and 20 weeks after initiation in 2 independent mice (**A**). In a separate experiment (**B**), treatment with MSU-42011 + carboplatin and paclitaxel (C/P) was started eight weeks after initiation, and ultrasound images of the same lung tumors were obtained 11, 15 and 20 weeks after initiation (or after 3, 7 and 12 weeks of treatment). Scale bar = 1 mm.

We have previously demonstrated efficacy of rexinoids in a model of Kras-driven lung cancer^17,21,46^, but improvements in imaging technologies now allow the non-invasive detection and visualization of lung tumors in living mice^47,48^. The Vevo 2100 high-frequency ultrasound provides axial resolution down to 30 µm. Using this approach, we are able to visualize tumors on the surface of the lungs in living mice. In order to follow the progression of lung tumors over time in our model, A/J mice were injected with the carcinogen vinyl carbamate, which induces *Kras* mutations and initiates lung carcinogenesis^21,49–51^. As shown in Fig. 2A, lung tumors as small as 0.4 mm in diameter can be detected by ultrasound by 8 weeks after initiation. The subpleural tumors displayed as areas of reduced echogenicity with hyperechogenic borders and posterior echo enhancement. Both the number and size of detectable lung tumors increase over time, as shown in 2 different mice at 8, 12 and 20 weeks after initiation (Fig. 2).

To determine if ultrasound could detect differences in tumor size and progression during treatment, A/J mice were challenged with vinyl carbamate. Eight weeks later, when lung tumors could be detected by ultrasound (Fig. 2A), mice were fed control diet or MSU-42011 in combination with carboplatin and paclitaxel (C/P). The same mouse in each group was imaged at 11, 15 and 20 weeks after initiation, which is after 3, 7 and 12 weeks of treatment. As shown in Fig. 2B, the number and size of detected lung surface tumors increased in the control group but not in the treatment group.

### MSU-42011 reduces lung tumor burden in A/J mice

**Figure 3:**
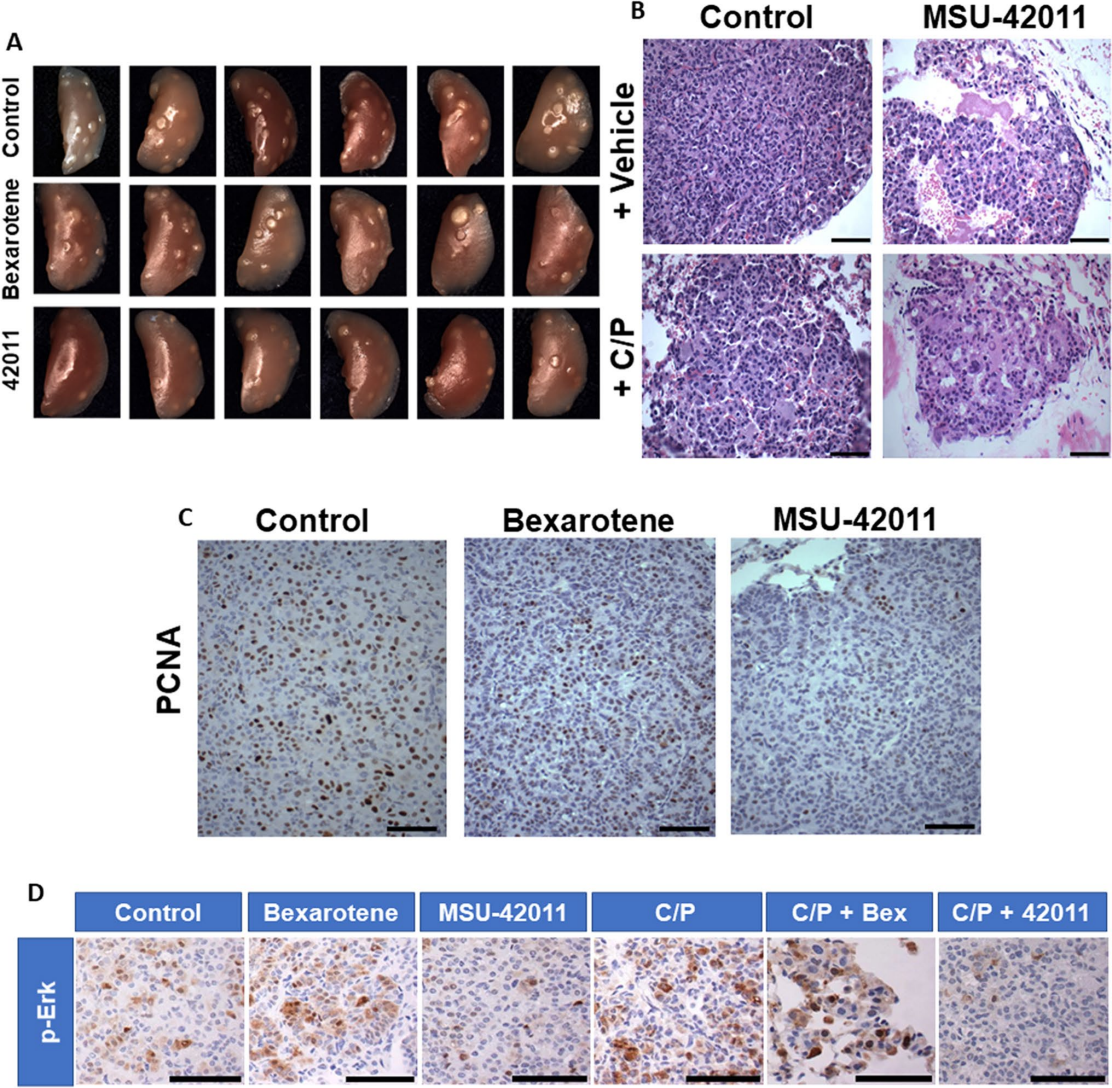
MSU-42011 alone decreases tumor burden and induces beneficial changes in tumor structure. Vinyl carbamate was used to initiate lung carcinogenesis in A/J mice. Starting 8 weeks after initiation, mice were fed experimental diets containing rexinoids (100 mg/kg diet), alone or in combination with carboplatin and paclitaxel (C/P) as described in Table 1. After 12 weeks of treatment, lungs were harvested and representative pictures (**A**, 8X magnification) of the left lungs from each group are shown. Changes in the histological architecture of the tumor are shown by H & E staining of tumor sections (**B**; scale bar = 60 microns) and proliferation of cells or Ras signaling within the tumor was analyzed by immunohistochemistry for proliferating cell nuclear antigen (**C**) or p-ERK expression (**D**), respectively**.** Scale bar = 60 microns.

The entire cohort of mice, initiated as described above, were sacrificed after 12 weeks of treatment with rexinoids, alone and in combination with chemotherapy. Lungs were harvested and the identity of the treatment group blinded prior to analysis. Mice treated with MSU-42011 alone had markedly fewer tumors upon gross observation of lungs compared to the bexarotene and control groups (Fig. 3A). These results were confirmed on tumor sections, as MSU-42011 significantly (*p* < 0.05) decreased tumor number, size, and overall tumor burden per slide (Table 2 and Supplemental Fig. 2). The average number and tumor burden, respectively, were 2.7 ± 0.3 and 0.43 ± 0.08 mm^3^ in the MSU-42011 group vs. 4.25 ± 0.4 and 0.93 ± 0.09 mm^3^ in the control group, a 36–38% decrease (*p* < 0.05). MSU-42011 was superior to bexarotene, as bexarotene was inactive as a single agent in this model. None of these parameters were significantly different in the bexarotene group compared to the control group. The average tumor size was significantly (*p* < 0.05) lower in lung sections from mice treated with MSU-42011 compared to the bexarotene group, and treatment with MSU-42011 alone (0.43 ± 0.08 mm^3^) decreased overall tumor burden (*p* = 0.076) compared to mice treated with bexarotene alone (0.72 ± 0.2 mm^3^).

**Table 2:** MSU-42011 decreases the number and size of lung tumors when used to treat established lung tumors in A/J mice.

	Control	Bexarotene	MSU-42011	C/P	Bex + C/P	42011 + C/P
**Tumor #, size, and burden**						
# of slides	28	30	30	24	28	24
Average # tumors/slide (% control)	4.25 ± 0.4 (100%)	3.37 ± 0.3 (79%)	2.7 ± 0.3*^$^ (64%)	1.75 ± 0.3*^ɸ^ (41%)	1.89 ± 0.3*^ɸ^ (45%)	1.42 ± 0.2*^ɸ^ (33%)
Average tumor size, mm^3^ (% control)	0.22 ± 0.02 (100%)	0.22 ± 0.04 (100%)	0.16 ± 0.02*^ɸ^ (73%)	0.08 ± 0.02*^ɸ†^ (36%)	0.05 ± 0.01*^ɸ†^ (22%)	0.05 ± 0.01*^ɸ†^ (24%)
Average tumor burden, mm^3^ (% control)	0.93 ± 0.09 (100%)	0.72 ± 0.2^#^ (78%)	0.43 ± 0.08* (62%)	0.14 ± 0.04*^ɸ†^ (15%)	0.09 ± 0.02*^ɸ†^ (10%)	0.07 ± 0.02*^ɸ†^ (8%)
**Tumor histopathology**						
# Low grade tumors (% total)	2 (2%)	1 (1%)	7 (9%)*	1 (2%)	0	1 (3%)
# Medium grade (% total)	25 (21%)	29 (29%)	24 (29%)	21 (50%)^!^	28 (53%)^!ɸ^	20 (59%)^!†^
# High grade tumors (% total)	92 (77%)	71 (70%)	50 (62%)*	20 (48%)^!^	25 (47%)^!ɸ^	13 (38%)^!†^

Female A/J mice were injected with 2 doses of vinyl carbamate (0.32 mg/injection), 1 week apart, to initiate lung carcinogenesis. After 8 weeks, mice were fed either control AIN-93G diet or rexinoids in the same diet (100 mg/kg diet or ~ 25 mg/kg body weight). One week after diets started, mice were injected i.p. every other week for a total of 6 injections with carboplatin (C—50 mg/kg) and paclitaxel (P—15 m/kg). After 12 weeks on diet, lungs were harvested and processed for sectioning and H & E staining. Values shown are mean ± SE. *, *p* < 0.05 vs. control; ɸ, *p* < 0.05 vs. bexarotene; †, *p* < 0.05 vs. MSU-42011; #, *p* = 0.076 vs. 42011; !, *p* < 0.001 vs. control; $, *p* = 0.114 vs bexarotene.

The carboplatin/paclitaxel (C/P) treatment group significantly (*p* < 0.05) reduced tumor number, size and burden in all groups compared to the groups receiving either control diet alone or a rexinoid diet alone (Table 2, Supplemental Fig. 2). However, the trend in these data showed that the combination of MSU-42011 + C/P was the most effective in reducing tumor number (67% reduction compared to the control group), tumor size (76% reduction), and overall tumor burden (92% reduction compared to the controls). Because the chemotherapy regimen was more effective than anticipated, this trend did not reach statistical significance.

### Superior toxicity profile of MSU-42011 compared to bexarotene

Mouse weights were monitored as a marker of drug toxicity (Supplemental Fig. 3). The rexinoids were well-tolerated when fed in diet, as the mice in the control, bexarotene, and MSU-42011 groups all gained weight as expected during the study. There were no significant differences in the average weights between groups of mice on single drugs. However, average mouse weights were significantly (*p* < 0.05) lower in all groups treated with chemotherapy (C/P) compared to groups treated with rexinoids or control diet alone (Supplemental Fig. 3).

Because of the known side effect of hypertriglyceridemia induced by bexarotene, triglyceride and cholesterol levels were measured in plasma collected from mice at the end of the study (Table 3). As shown in Table 3, plasma triglycerides (2.21 ± 0.16 nmol/µL) and cholesterol levels (1.99 ± 0.1 µg/µL) were significantly (*p* < 0.01) higher in mice treated with bexarotene compared to the controls (1.32 ± 12 nmol/µL and 1.26 ± 0.07 µg/µL, respectively. In contrast, mice treated with MSU-42011 had significantly (*p* < 0.01) lower levels of plasma triglycerides and cholesterol (1.39 ± 0.11 nmol/µL and 1.4 ± 0.05 µg/µL, respectively) compared to bexarotene. There were no differences in plasma levels of triglycerides or cholesterol between MSU-42011-treated mice and the controls (Table 3). These results confirm the results from the in vitro SREBP assays with MSU-42011 (Table 1, Fig. 1D). There were no significant differences in the drug levels in the plasma of mice fed MSU-42011 (97.4 ± 13.3 nM) compared to mice fed bexarotene (81.2 ± 14.9 nM) when measured by LC–MS, confirming similar bioavailability when given in diet (Table 3).

**Table 3:** Drug levels of rexinoids and cholesterol and triglyceride levels in the plasma of A/J mice.

	Control	Bexarotene	MSU 42011
Drug levels (nM)	None	81.2 ± 14.9	97.4 ± 13.3
Cholesterol (µg/µL)	1.26 ± 0.07	1.99 ± 0.1ǂ	1.4 ± 0.05*
Triglycerides (nmol/µL)	1.32 ± 0.12	2.21 ± 0.16ǂ	1.39 ± 0.11*

Female A/J mice were fed rexinoids (100 mg/kg diet) or control diet for 12 weeks. Steady state drug levels in plasma were determined by liquid chromatography-mass spectrometry. Cholesterol and triglycerides levels were quantified using commercially available kits as described in the Methods. n = 8 mice/group. ǂ, *p* < 0.01 vs. control; *, = *p* < 0.01 vs. bexarotene. Results shown are mean ± SE.

### MSU-42011 changes the histological characteristics of lung tumors in A/J mice

At the end of the treatment study, lungs were harvested, sectioned, stained by H&E and randomized to blind the identity of the treatment group until all slides had been analyzed. Tumors were graded as described^46,52^ for nuclear and histological severity and given a grade of either low, medium, or high. Tumors assigned a low nuclear grade have smaller, more regular nuclei, while tumors in the high nuclear grade have enlarged, irregular nuclei with visible chromatin condensation and mitotic figures. Tumors in the low histological grade have space between cells, while tumors with a high histological grade are dense and obliterative, with very little to no space between tumor cells. In mice treated with MSU-42011 alone, 9% of the tumors were low grade, which was significantly higher (*p* < 0.05) than the 2% of observed tumors which were low grade in the control group (Table 2). Furthermore, mice treated with MSU-42011 had significantly (*p* < 0.05) fewer high grade tumors (62% of all tumors) compared to the control group (77% of all tumors). There were no differences in tumor grades between bexarotene-treated mice and the controls. Importantly, MSU-42011 induced a notable change in the histological organization of tumors characterized by a loss of cells and increased acellular space within the tumor borders (Fig. 3B). Immunohistochemical (IHC) staining for cleaved caspase 3 showed no difference between the groups (Supplemental Fig. 4), as the malignant cells had likely already been cleared out of the tumor borders at the time of analysis. However, IHC for proliferating cell nuclear antigen (PCNA), a marker for cell proliferation, revealed that tumors treated with MSU-42011 contained fewer actively proliferating cells compared to the control and bexarotene groups (Fig. 3C). Additionally, p-ERK was markedly decreased in tumors treated with MSU-42011 alone compared to tumors in the control and bexarotene groups (Fig. 3D), indicating that MSU-42011, but not bexarotene, decreased proteins downstream of Ras within tumors. A similar trend was observed in the groups co-treated with carboplatin and paclitaxel, as p-ERK was visibly reduced in tumors treated with MSU-42011 + C/P compared to tumors treated with control + C/P or bexarotene + C/P.

### The combination of MSU-42011 and chemotherapy alters immune cells within the tumor microenvironment

Rexinoids have limited effects on the proliferation of various cancer cells in vitro^9^, and MSU-42011 had no effect on the growth of A549 lung cancer cells (Supplemental Fig. 5). We have shown that treatment with the rexinoid LG100268, but not bexarotene, decreased the number of immunosuppressive myeloid-derived suppressor cells and tumor-promoting macrophages in the lung cancer model^21^ and in the MMTV-Neu mouse model of HER2+ breast cancer^53^. Therefore, we hypothesized that the effects of rexinoids observed in vivo occur through modulation of immune cells within the tumor microenvironment. As *Kras* mutations increase pro-inflammatory pathways that alter the tumor microenvironment and contribute to tumor progression^54^, we used flow cytometry (gating strategy shown in Supplemental Fig. 6) and IHC to investigate immune cell populations in the lungs of the A/J mice treated with rexinoids.

**Figure 4:**
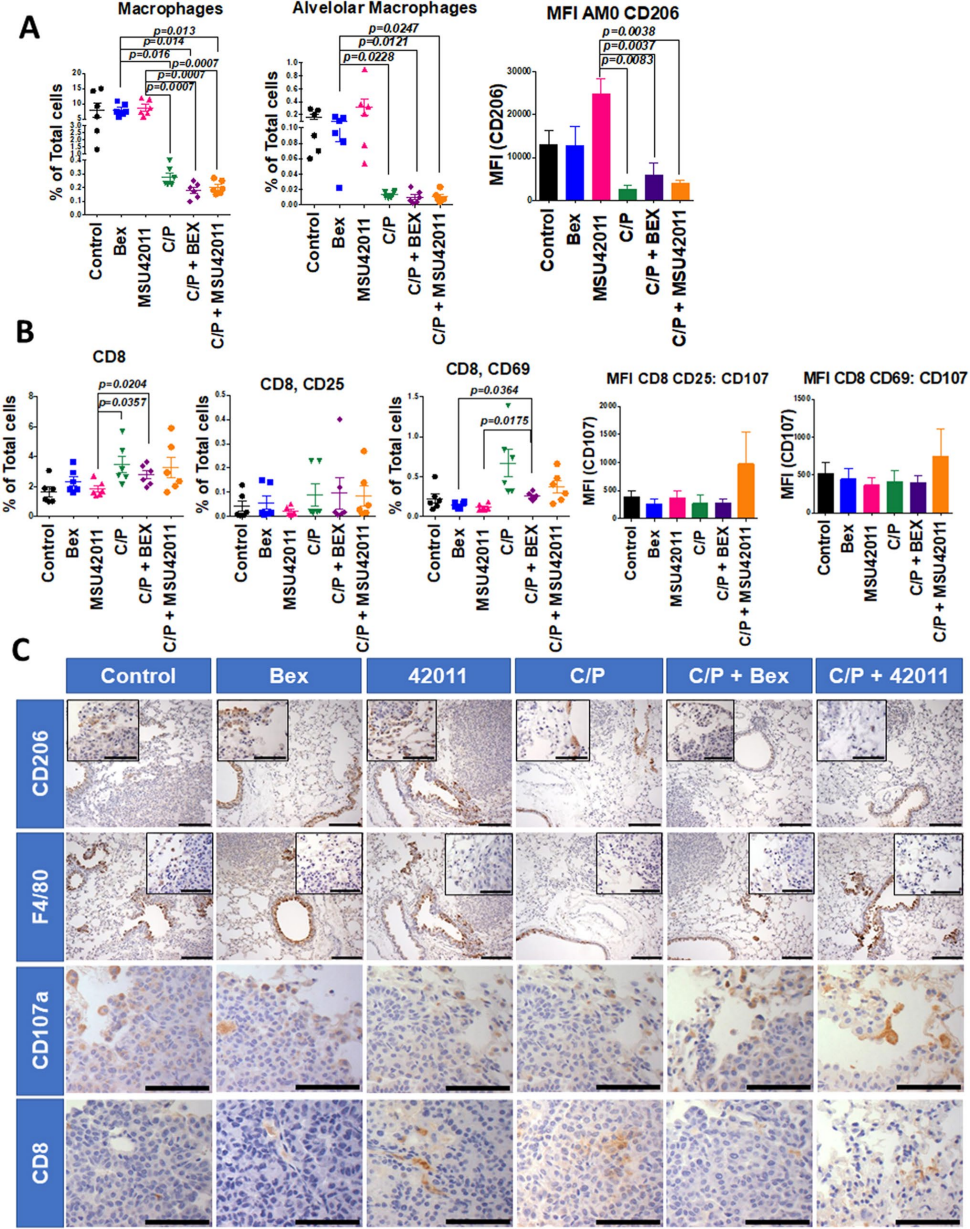
The combination of MSU-42011 and carboplatin/paclitaxel changes the immune microenvironment in tumor-bearing lungs. A/J mice with established lung tumors were treated with control diet, rexinoids in diet (100 mg/kg diet), 6 total doses of carboplatin (C—50 mg/kg) and paclitaxel (P—15 m/kg), given every other week, or the combination for 12 weeks. Flow cytometry (A and B) and immunohistochemistry (C) were used to detect immune populations in the lung. (**A**) Levels of macrophages (CD45^+^, CD11b^+^, Gr1-, CD64^+^, ia-ie^+^) and alveolar macrophages (CD45^+^, CD11b^+^, CD11c^+^, Gr1-, CD64^+^, ia-ie^+^) in the lung and mean florescence for CD206 (M2 macrophage marker) in alveolar macrophages. (**B**) Expression of CD8 cytotoxic T cells, the T cell activation markers CD69 and CD25, and the degranulation marker CD107a in the lung. The mean florescence for early (CD69) and late (CD25) activated cytotoxic CD8 T cells are also shown. (**C**) Immunohistochemistry for F4/80 (pan macrophage marker), CD206 M2 macrophages, CD107a degranulation, and CD8 T cells in the lung. Scale bar represents 60 μM.

RXR and its nuclear receptor partners are known to regulate migration and activity of macrophages and other immune cells^55^. No differences were observed in the percentage of total (CD45 +) immune cells or CD3 + T cells in the lung (Supplemental Fig. 7). While total and alveolar macrophages in the lungs were significantly lower in mice treated with the combination of MSU-42011 and C/P, treatment with MSU-42011 alone did not alter these macrophage populations in comparison to the controls (Fig. 4A). However, treatment with the combination of MSU-42011 and C/P was associated with a decrease in CD206, a marker of tumor-promoting macrophages, on alveolar macrophages as measured by mean fluorescence intensity. This decrease was statistically significant (*p* = 0.0038) in comparison to treatment with MSU-42011 alone. The flow cytometry results were confirmed by IHC staining for F4/80 (a general marker for macrophages) and CD206. Alveolar macrophages promote tumor growth and are sensitive to chemotherapy^56^. Combination treatment with MSU-42011 and C/P also increased infiltration of CD8 cytotoxic T cells into the lung and increased activation of these cells, as shown by increased expression of CD69^57^. Consistent with the infiltration and activation of CD8 T cells, expression of CD107a, a marker of degranulation in cytotoxic immune cells, also increased on activated CD8 cytotoxic lymphocytes (both CD25 and CD69 positive) in the lungs (Fig. 4B, C).

## Discussion

Despite the promising preclinical studies with rexinoids such as LG100268 and IRX194204^9,20^, side effects and a difficult development pathway (expired patents) have hindered clinical development of these potent and selective rexinoids. To address these challenges, we have synthesized 23 novel compounds and identified MSU-42011 as a lead compound. This new rexinoid inhibited the production of NO in macrophage-like cells without elevating SREBP in vitro*.* Because bexarotene was most effective clinically in lung cancer patients with elevated triglycerides^13,14^, it was possible that a rexinoid such as MSU-42011 that did not elevate triglyceride levels in vivo (Table 3) would lack anti-tumor activity*.* However, MSU-42011 had better single agent activity than bexarotene for treating established Kras-driven lung adenocarcinomas. Our data and other studies^44,58,59^ confirm that these biological activities can be separated.

The majority of preclinical studies have tested rexinoids in models of breast cancer^20^. The few studies investigating rexinoids for treatment of lung cancer used xenograft models or the carcinogen NNK to induce lung cancer^60–62^. However, xenograft models lack a functioning immune system and although NNK is found in cigarettes, it induces adenomas, which are far less aggressive than the adenocarcinomas induced by vinyl carbamate in A/J mice^41^. Thus, these models are less representative of human KRAS-driven NSCLC. Even though new inhibitors target aberrant RAS activity^63,64^, no drugs are currently approved for tumors driven by *KRAS* mutations, making the results of this study particularly relevant.

In addition to the efficacy of MSU-42011 in lung cancer, we have previously demonstrated the efficacy of rexinoids in a model of pancreatic ductal adenocarcinoma driven by an activating Kras^G12D^ mutation^65^ and in the MMTV-Neu model of HER2 + breast cancer^53^, in which Ras activity is high^66–68^. These studies suggest that rexinoids may be particularly effective in the treatment of RAS-driven tumors. Consistent with these findings, MSU-42011 also reduces p-ERK expression, a downstream effector of RAS activation, in tumors (Fig. 3D). While the development of novel therapeutics for KRAS-driven NSCLC is undeniably critical, rexinoids should be tested in lung cancer models harboring different mutations found in human lung cancer. *EGFR*-mutated lung cancers are especially relevant, as EGFR activation can activate RAS signaling, and patients with *EGFR* mutations may have a concomitant gain-of-function *KRAS* mutation^69^.

Proinflammatory pathways are upregulated in *KRAS*-dependent tumors, including increased production of the chemokines IL-1β, CCL7, and CCL2, as a result of crosstalk between *KRAS*-mutant tumor cells and host myeloid cells^54^. Inflammation is important in all stages of carcinogenesis, as it contributes to genomic instability, malignant cell proliferation, stimulation of angiogenesis, and metastatic dissemination^70^. Induction of proinflammatory chemokines leads to the recruitment of immune cells which modulate the tumor microenvironment. Our results suggest a potential role for resident alveolar macrophages in lung cancer progression, which is supported by studies describing the importance of RXR in the differentiation of macrophages^71,72^. Macrophages make up a substantial portion of immune cells infiltrating into the tumor microenvironment and play a key role in tumorigenesis and disease progression^73^. RXR is also important in other inflammatory responses, including regulation of Th1/Th2 responses by dendritic cells through the adaptive immune response^74^. Additional studies are needed to fully understand the immunomodulatory effects of MSU-42011 and other rexinoids within the tumor microenvironment and to investigate if immune cells are required for the anti-cancer activity of rexinoids.

While the use of ultrasound to detect the onset and development of tumors in mouse models such as the KPC mouse model of pancreatic cancer is common^75^, using ultrasound imaging to monitor lung tumors in a living animal and follow them over time is not widely used. It is more challenging to image tumors in the lungs of mice because of the inability of ultrasound to penetrate air within lungs or to penetrate through ribs and sternum, as well as to accommodate motion created by the rapid beating of the heart. Although it is easier to detect tumors on the periphery of the lung than all of the tumors within the lung, ultrasound and other imaging techniques are valuable, but under-utilized tools for monitoring efficacy in drug development studies^48,76^. This approach provides valuable qualitative observations which can be used not only to monitor treatment efficacy and disease progression throughout the study but also to supplement end-of-life measurements taken at the conclusion of the study.

In summary, we have synthesized and screened 23 novel rexinoids. Our results confirm that iNOS and SREBP activity are distinct, and the in vivo results match the predictions of the in vitro screening assays. Trends in cholesterol/triglyceride levels corroborate the SREBP assays, and the in vivo anti-tumor efficacy complements results from the iNOS assays. Our data suggest that MSU-42011, unlike bexarotene, is active as a single agent for treating Kras-driven lung cancer and hinders the aggressive phenotype seen in these tumors. Work is continuing to fine tune these observations, to discover new analogs of higher potency and selectivity, and to evaluate the efficacy of MSU-42011 when combined with immunotherapy.

## Methods

### Chemistry

Description of the synthesis of all compounds is described in Supplemental Data.

### Biology-cell culture

HepG2 human liver hepatocellular carcinoma cells, RAW264.7 murine macrophage-like cells, and A549 human lung adenocarcinoma cells were purchased from ATCC. They were cultured at 37 °C and 5% CO2 in RPMI1640, DMEM or F-12 K media, respectively, all supplemented with 10% FBS and 1% penicillin/streptomycin. Media and supplements were purchased from Corning Cellgro (Mediatech, Manassas, VA).

### iNOS inhibition assay

RAW264.7 cells were plated in 96-well plates (20,000 cells/well). After overnight attachment, cells were treated with twofold serial dilutions (0–1000 nM) of rexinoids. After a 20 min incubation at 37 °C, cells were stimulated with 1 ng/mL LPS for 24 h. NO production was measured in culture medium using the Griess reaction. IC_50_ values were calculated using GraphPad Prism 8.1.2 software for Windows using a log([nM drug concentration]) vs. response curve fit.

### SREBP expression assays

HepG2 were plated in 6-well plates (1 × 10^6^ cells/well) and, after allowing the cells to attach overnight, were treated with 300 nM rexinoids and/or varying concentrations of T0901317 (TOCRIS # 2373), GSK 2033 (TOCRIS # 5694), HX 531 (TOCRIS # 3912) for 8 h. Total RNA was isolated with TRIzol (Invitrogen, Carlsbad, CA) and 2 µg RNA was used to synthesize cDNA using the SuperScript III reverse transcriptase kit (Invitrogen, Carlsbad, CA). Primers (IDT) for SREBP (F: 5′-GCCGGTTGATAGGCAGCTT-3′; R: 5′-GGTGAGTGGCGGAACCATT-3′) and GAPDH (F: 5′-GGAGCGAGATCCCTCCAAAAT-3′; R: 5′-GGCTGTTGTCATACTTCTCATGG-3′), the SYBR Green Supermix (Bio-rad Laboratories, Hercules, CA) and the QuantStudio 7 Flex Real-Time PCR system (95 °C for 20 s followed by 40 cycles of 95 °C for 15 s and 60 °C for 1 min were used to detect mRNA expression. Relative SREBP/GAPDH gene expression was calculated by the delta-delta Ct method^21^. To evaluate protein expression by western blotting, HepG2 cells were plated as above and treated with 300 nM of rexinoids for 24 h. Cells were harvested and lysed in RIPA buffer (1 M Tris–Cl, 5 M NaCl, pH 7.4, 0.5 M EDTA, 25 mM sodium deoxycholate, 1% triton-X, 0.1% SDS, 0.0125% aprotinin) with protease inhibitors (1% PMSF/0.5% apoprotein/0.1%leupeptin). Protein concentration was measured using a BCA assay (Sigma-Aldrich). Proteins (10 µg/well) were separated in 10% SDS-PAGE gels and transferred to nitrocellulose membranes. Membranes were incubated with SREBP (Active Motif; 1:1000) and GAPDH (Santa Cruz Biotechnology; 1:8000) primary antibodies followed by anti-rabbit secondary antibodies conjugated to horseradish peroxidase (Cell Signaling). Signal was detected using the ECL Western Blotting Substrate (GE Healthcare), and protein levels quantified using ImageJ.

### RXR activation assay

RXR activation was measured using a RXRα reporter assay kit (Indigo Biosciences) following the manufacturer’s protocol. Cells (triplicate wells/concentration) were treated with fourfold dilutions (0–5000 nM) of rexinoids for 24 h, and luminescence intensity detected using a Biotech Synergy Neo fluorescent plate reader. EC_50_ values were calculated using GraphPad Prism 8.1.2 software for Windows using a log([M drug concentration]) vs. response curve fit.

### Treatment of lung adenocarcinomas in vivo

All experiments were performed ethically in accordance with the best practices and AAALAC-accredited Standards for the Management of Laboratory Animals at Michigan State University (MSU). These standards include making every effort to minimize suffering. Mice were euthanized by inhalation of carbon dioxide followed by removal of the lungs. All animal protocols were approved by the Institutional Animal Care and Use Committee (IACUC, protocol 201,800,050)) at MSU. At 7 and 8 weeks of age, A/J female mice (Jackson Laboratories) were injected i.p. with vinyl carbamate (Toronto Research Chemicals, 0.32 mg/mouse). Mice were fed a semi-synthetic diet (AIN-93G BioServ, Flemington NJ), beginning one week before initiation with vinyl carbamate and then for 8 weeks after the first injection. The mice were then randomized into groups (n = 15 mice/group) and fed treatment diets (100 mg rexinoid/kg AIN-93G diet or ~ 25 mg/kg body weight) for 12 weeks. Rexinoids were dissolved in a vehicle of 12.5 ml ethanol and 37.5 ml Neobee oil (Spectrum Chemical, Gardena CA) per kg AIN-93G diet. Six total doses of paclitaxel (Fisher; 15 mg/kg i.p.) and then carboplatin (Fisher; 50 mg/kg i.p.) were given every other week, beginning 9 weeks after injection with vinyl carbamate. After 12 weeks on the treatment diets, blood and lungs were harvested. Lungs were inflated with PBS and surface lesions counted. The left lung was fixed in neutral-buffered formalin (NBF), then sample numbers blinded and randomized. Grossly visible lung tumors on the surface of the left lung were counted. The left lung was embedded in paraffin, step-sectioned (6 sections/lung, 200 microns apart) and then stained with Haemotoxylin and Eosin (H&E). The number, size, and histopathology of tumors on the slides were then evaluated as described^77^. The right lobes of the lung were divided into halves. One half was analyzed by flow cytometry and the other half flash frozen (CO_2_ and ethanol bath) and stored at − 80 °C.

### Ultrasound

At 11, 15, and 20 weeks after initiation, anesthesia was induced using 2–4% isofluorane in oxygen at 0.8–2 L/min in a chamber and then maintained using 1.5–2% isofluorane in oxygen at 0.8 L/min using a nosecone. Animals were kept on a heating pad or a heated platform while under anesthesia. Fur was removed from the thoracic area using a chemical depilitory (Nair) immediately prior to imaging. A Vevo 2100 (Fujifilm VisualSonics) high-frequency ultrasound with a MS550D 40 MHz transducer was used to image lung tumors.

### Flow cytometry

The same two lobes of the right lung were harvested from each A/J mouse (n = 6/group) for flow cytometry and incubated in digestion media consisting of collagenase (300 U/ml, Sigma), dispase (1 U/ml, Worthington), and DNAse (2 U/ml, Calbiochem) for 30 min at 37 °C with stirring. Cells were then passed through a 40 µm cell strainer (BD Falcon), and red blood cells eliminated with lysis solution. Single cells were resuspended in a solution of Brilliant buffer (BD Bioscience) and stained for 30 min at 4 °C with the following antibodies: CD45-Brilliant violet 510 (30F11), CD24-Brilliant violet 604 (M1/69), CD64-Brilliant violet 711 (X54-5/7.1), AI/AE- Brilliant violet 650 (M5/114.15.2), CD8-Alexafluor 700 (53–6.7), CD4-FITC (GK1.5), LyC6/LyG6-APC (rb6-8C5), CD11b-PE-Cy7 (M1/70), CD11c-PE-Cy5 (N418), CD206- Brilliant violet 421 (C068C2), CD69- Brilliant violet 785 (41.2F3), CD25-PE (3C7), CD107a-Alexafluor 647 (1D4B), and 5 μg/ml anti-mouse CD16/CD32 antibody (Biolegend) to reduce antibody binding to Fc receptors. Zombie NIR staining was used to exclude dead cells. Cells were analyzed using a Cytek Aurora equipped with 5 lasers (UV 355 nm, violet 405 nm, blue 488 nm, yellow-green 561 nm, and red 640 nm) and FlowJo x.10.0.7r2 software (Tree Star). The gating strategy used and shown in Supplemental Fig. 6 was adapted from a published protocol^78^.

### Immunohistochemistry

Paraffin blocks of the left lobe of the lung were sectioned, and hydrogen peroxide was used to quench endogenous peroxidase activity. Sections were immunostained with antibodies against CD206 (1:200, Abcam), CD8 (1:40, 53–6.7, Biolegend), cleaved caspase 3 (1:100, 5A1E, Cell Signaling), PCNA (1:200, sc-56, Santa Cruz Biothechonlogies), F4/80 (1:50, BM8, Invitrogen), CD107a (1:50, Biolegend), p-ERK (1:50, Cell Signaling) and visualized with biotinylated anti-rabbit or anti-rat secondary antibodies (Cell Signaling or Vector Labs), as previously described^53^. Signal was detected using a DAB substrate (Cell Signaling) following the manufacturer’s recommendations. Sections were counterstained with hematoxylin (Vector Labs).

#### HPLC–MS

Blood was collected via cardiac puncture into 1 ml lithium heparin tubes (Greiner Bio-one). Blood was centrifuged at 5000 g for 5 min at 4 °C. Plasma was separated and frozen at − 80 °C until processed as described^79^. Acetonitrile was added to precipitate plasma proteins. Samples were centrifuged at 14,000 g for 5 min, and the supernatant was filtered with a 0.2 µm syringe filter. Each sample was dried overnight and reconstituted in 200 µl of 50:50 methanol:water solution for analysis via HPLC. HPLC was performed on a Quatro Premier instrument with an Ascentis Express C18 column and an Acquity UPLC system. The mobile phase A consisted of 10 mM aq. ammonium acetate and the mobile phase B consisted of methanol. The following gradient was applied 99–5% A (0–4 min), 5% A (4–5.1 min), 5–99% A (5.1–6 min). The flow rate was 0.3 ml/minute, and bexarotene and MSU-42011 were detected at ~ 4.5 min. A standard curve was prepared by processing plasma from untreated animals and spiking the samples with 0.1–3 μM of the appropriate rexinoid then reconstituting the samples.

### Lipid levels in plasma

Triglyceride and cholesterol levels in plasma (n = 8 mice/group) harvested at necropsy were measured using the Triglyceride Quantification Assay Kit (Abcam) or the Cholesterol Quantification Kit (Sigma-Aldrich), respectively, using the recommended protocol supplied by the manufacturer.

### Statistical analysis

In vitro experiments were performed in triplicate (MTT assay) or quintuplet (iNOS suppression assay) for each concentration of drug, and independent experiments were repeated a minimum of three times. Results were expressed as the mean ± standard deviation or the mean ± standard error as indicated in the respective figure/table legends. Data from in vitro experiments fit normal distributions and were analyzed using one-way ANOVA, and significant differences between groups were analyzed by the Tukey HSD multiple comparison method (VassarStats.com). Data from in vivo experiments (SigmaStat 3.5) were analyzed by one-way ANOVA followed by the Holm-Sidak test for multiple comparisons when data fit a normal distribution, and the Kruskal–Wallis one-way ANOVA on ranks followed by the Dunn test for multiple comparisons when the data did not pass the normality test. McNemar’s Z test was used to compare differences in the histopathological grades of lung tumors. *p* < 0.05 was considered statistically significant.

## Supplementary information

Supplementary Information

Supplementary Information
