# Supplementary material for: The novel rexinoid MSU-42011 is effective for the treatment of preclinical Kras-driven lung cancer
Source: Sci Rep. 2020 Dec 17;10:22244. doi: 10.1038/s41598-020-79260-8 (PMC7746742; doi:10.1038/s41598-020-79260-8)
Supplement: Supplementary file 1 — Supplementary Information [file 41598_2020_79260_MOESM1_ESM.docx]

**The novel rexinoid MSU-42011 is effective for the treatment of preclinical Kras-driven lung cancer**

Jessica A. Moerland^1^, Di Zhang^1^, Lyndsey A. Reich^1^, Sarah Carapellucci^1^, Beth Lockwood^1^, Ana S. Leal^1^, Teresa Krieger-Burke^1,2^, Bilal Aleiwi^1,3^, Edmund Ellsworth^1,3^, Karen T. Liby^1^

Authors’ affiliations: ^1^Department of Pharmacology & Toxicology, ^2^In Vivo Facility, ^3^Medicial Chemistry Core, Michigan State University, East Lansing, MI

**Corresponding author:** Karen T. Liby, Michigan State University, Department of Pharmacology and Toxicology, B430 Life Science Building, 1355 Bogue Street, East Lansing, MI 48824. Phone: 517-884-8955; Fax: 517-353-8915; Email: libykare@msu.edu

**Key Words:** Rexinoid, RXR, bexarotene, lung cancer, Kras, A/J mouse model, ultrasound, nitric oxide, SREBP, immune modulation

**Chemistry**

**General Information**. All commercial reagents and solvents were used as received without further purification. All reactions were monitored with thin layer chromatography (TLC) using Silica gel 60G F254 TLC plates or Advion Avant A-2041 LCMS system. Filtration was performed through silica gel (0.04–0.07 mm; 215–400 mesh ASTM). Flash column chromatography (FCC) was made with a Teledyne ISCO High-Performance Flash Chromatography using a 0.1 mm path length flow cell UV detector/recorder module. 1H and 13C NMR spectra were recorded on an Agilent VXR (500 and 125 MHz, respectively) solvent peak as an internal standard. 1H chemical shifts are referenced to residual non-deuterated solvent (CHCl3, dH = 7.26 ppm). 13C chemical shifts are referenced to the solvent signal (CDCl3, dC = 77.16 ppm). Spectral data are reported as follows: chemical shift (as ppm referenced to tetramethylsilane), multiplicity (s = singlet, d = doublet, dd = double doublets, dt = double triplet, t = triplet, q = quartet, m = multiplet, br = broad peak), coupling constants J values are quoted in Hertz (Hz). High resolution mass spectra were recorded with a Waters G2-XS QTof using electrospray positive ionization. Melting points were recorded on an electrothermal instrument and were not corrected.

**MSU-41404**

**Methyl 4-(3,8,8-trimethyl-5,6,7,8-tetrahydronaphthalene-2-carbonyl)benzoate.** A stirred slurry of 4-(methoxycarbonyl)benzoic acid (0.80 g, 4.4 mmol) in dichloromethane (12.0 mL) was treated with oxalyl chloride (0.57 mL, 6,6 mmol), 3 drops of dimethylformamide and stirred for 30 minutes to provide a homogeneous solution, which was concentrated in vacuo. The resulting residue was then re-dissolved in dichloromethane (10.0 mL) and the solution again concentrated in vacuo, this process repeated 3 times. The residue was resuspended in dichloromethane (10.0 mL) and treated with a solution of 1,1,6-trimethyl-1,2,3,4-tetrahydronaphthalene (Fondekar, K. P. P. et al. Natural Product Communications, 7(7), 827-830; 2012.) (1.0 g, 4.0 mmol) in dichloromethane (5.0 mL) and treated with aluminum trichloride (1.2 g, 10.0 mmol) at 0^o^C, allowed to warm and the mixture stirred overnight at room temperature. The mixture was then treated with 20% hydrochloric acid in ice and stirred overnight then diluted with additional dichloromethane and washed with 2.0 N NaOH. The organic layer was subsequently dried with sodium sulfate, filtered and concentrated in vacuo. The resulting residue was then purified via MPLC (SiO_2_, 100% hexanes to 10% ethyl acetate / hexanes to provide an oil (1.0 g). ^1^H NMR (500 MHz, Chloroform-*d*) δ 8.12 (m, 2H), 7.85 (d, *J* = 9.0 Hz, 2H), 7.28 (s, 1H), 6.99 (s, 1H), 3.97 (s, 3H), 2.79 (t, *J* = 6.0 Hz, 2H), 2.31 (s, 3H), 1.83 – 1.82 (m, 2H), 1.69 – 1.67 (m, 2H), 1.22 (s, 6H).

**Methyl 4-[1-(3,8,8-trimethyl-5,6,7,8-tetrahydronaphthalen-2-yl)ethenyl]benzoate.** A slurry of methyltriphenylphosphanium bromide (0.63 g, 1.8 mmol) in tetrahydrofuran (5.0 mL) under a nitrogen atmosphere at -78^o^C was treated with a hexane solution (2.6 M) of n-butyllithium (0.67 mL, 1.8 mmol) via syringe. The mixture was then slowly warmed to 0^o^C and then recooled to -78 ^o^C and treated with a solution of methyl 4-(3,8,8-trimethyl-5,6,7,8-tetrahydronaphthalene-2-carbonyl)benzoate (0.30 g, 0.88 mmol) via cannula. The mixture was stirred for 1 hour at -78^o^C then allowed to slowly warm to ambient temperature, stirred for an additional hour then quenched with an aqueous saturated sodium chloride solution. The mixture was then extracted with hexames, this process repeated 3 times. The organic layers were combined, dried with sodium sulfate, filtered and the solvent removed in vacuo. The residue as then purified by MPLC (SiO2, 100% hexanes to 5% ethyl acetate / hexanes) to provide an oil (0.077 g). ^1^H NMR (500 MHz, Chloroform-*d*) δ 7.95 (dd, *J* = 8.0, 2.0 Hz, 2H), 7.35 (dd, *J* = 8.0, 2.0 Hz, 2H), 7.17 (s, 1H), 6.88 (s, 1H), 5.84 (d, *J* = 1.0 Hz, 1H), 5.31 (d, *J* = 2.0 Hz, 1H), 3.91 (s, 3H), 2.77 (t, *J* = 6.5 Hz, 2H), 1.93 (s, 3H), 1.85 – 1.81 (m, 2H), 1.70 – 1.68 (m, 2H), 1.29 (s, 6H).

**4-[1-(3,8,8-Trimethyl-5,6,7,8-tetrahydronaphthalen-2-yl)ethenyl]benzoic acid (MSU-41404).** A mixture of methyl 4-[1-(3,8,8-trimethyl-5,6,7,8-tetrahydronaphthalen-2-yl)ethenyl]benzoate (0.020 g, 0.050 mmol), potassium hydroxide (0.014 g, 0.25 mmol), methanol (0.50 mL) and water (0.50 mL) was heated to 80^o^C for 2 days. The mixture was cooled and acidified with 2.0 N hydrochloric acid and extracted with ethyl acetate (3 times). The organic layers were combined, dried with sodium sulfate, and the solvent removed in vacuo to provide a solid (0.021 g). ^1^H NMR (500 MHz, Chloroform-*d*) δ 8.10 – 7.97 (m, 2H), 7.45 – 7.34 (m, 2H), 7.18 (s, 1H), 6.89 (s, 1H), 5.86 (d, *J* = 1.3 Hz, 1H), 5.35 (d, *J* = 1.2 Hz, 1H), 2.78 (t, *J* = 6.3 Hz, 2H), 1.95 (s, 3H), 1.89 – 1.81 (m, 2H), 1.74 – 1.68 (m, 2H), 1.44 (s, 1H), 1.30 (s, 7H), 0.92 – 0.84 (m, 1H). HRMS (ES-) (M-H) Calc. 319.1706, Found 319.1703.

**MSU-41566**

**4-[1-(3,5-Di-tert-butylphenyl)-1-hydroxyethyl]benzonitrile.** A mixture of bromo-3,5-di-t-butylbenzene (0.60 g, 2.2 mmol), magnesium turnings (0.150 g, 6.25 mmol), iodine (2 crystals) in dry THF (5.0 mL) under a nitrogen atmosphere was heated to 60^o^C overnight then cooled to ambient temperature. 4-Acetylbenzonitrile (0.310 g, 2.12 mmol) was added and the mixture allowed to stir for 1 hour then quenched with aqueous solution of saturated ammonium chloride. The mixture was then extracted 3 times with ethylacetate, the organic layers combined, dried with sodium sulfate and the solvent removed in vacuo. The residue was then purified via MPLC (SiO_2_, 100% hexane to 15% hexanes /ethylacetate) to provide an oil (0.305 g, 43% yield). 1H NMR (500 MHz, Chloroform-d) δ 7.63 – 7.57 (m, 2H), 7.57 – 7.51 (m, 2H), 7.35 (t, J = 1.7 Hz, 1H), 7.26 (s, 1H), 7.22 (d, J = 1.8 Hz, 1H), 2.22 (s, 1H), 1.96 (s, 3H), 1.29 (d, J = 0.5 Hz, 18H).

**4-[1-(3,5-di-tert-butylphenyl)ethenyl]benzonitrile.** A mixture of 4-[1-(3,5-di-tert-butylphenyl)-1-hydroxyethyl]benzonitrile (0.305 g, 0.909 mmol) and p-toluene sulfonic acid (0.026 g, 0.14 mmol) in toluene (15 mL) in a 25 mL round-bottom flask equipped with Dean-Stark trap was heated to reflux for 24 hours, driving off the water. The mixture was then concentrated in vacuo and diluted with ethyl acetate then washed with water. The organic layer was then dried with sodium sulfate, filtered and concentrated in vacuo. The residue was then purified via MPLC (SiO_2_, 100% hexanes to 10% ethyl acetate / hexanes to provide a solid (0.25 g, 86% yield). 1H NMR (500 MHz, Chloroform-d) δ 7.65 – 7.59 (m, 2H), 7.49 – 7.44 (m, 2H), 7.42 (t, J = 1.9 Hz, 1H), 7.10 (d, J = 1.8 Hz, 2H), 5.55 (d, J = 14.9 Hz, 2H), 1.31 (s, 18H).

**4-[1-(3,5-di-tert-butylphenyl)ethenyl]benzoic acid (MSU-41566).** A mixture of 4-[1-(3,5-di-tert-butylphenyl)ethenyl]benzonitrile (0.050 g, 0.16 mmol), potassium hydroxide (0.089 g, 1.5 mmol), ethanol (5.0 mL) and water (1.0 mL) was heated to reflux for 14 hours. The mixture was then cooled, acidified with 1.0 N hydrochloric acid in water and then extracted 3 times with ethyl acetate. The organic layers were combined, dried with sodium sulfate and concentrated in vacuo to provide a solid (0.030 g, 60% yield). 1H NMR (500 MHz, Chloroform-d) δ 8.09-8.04 (m, 2H), 7.51 – 7.47 (m, 2H), 7.42 (s, 1H), 7.16 (s, 2H), 5.57 (s, 2H), 1.32 (s, 18H). HRMS (ES+) Calc. 336.209, Found 336.213.

**MSU-41405**

**Methyl 4-{1-[3-iso-propyl-4-(2-methylpropoxy)phenyl]-1-hydroxyethyl}benzoate.** A stirred slurry of 4-(methoxycarbonyl)benzoic acid (1.67 g, 9.27 mmol) in dichloromethane (10.0 mL) was treated with oxalyl chloride (1.20 mL, 13.91 mmol) and treated with 2 drops of dimethylformamide. The mixture was stirred for 30 minutes to provide a homogeneous solution, which was concentrated in vacuo. The resulting residue was then re-dissolved in dichloromethane (10.0 mL) and the solution again concentrated in vacuo, this process repeated 3 times. The residue was then again resuspended in dichloromethane (20.0 mL) and treated with a solution of 1-(2-methylpropoxy)-2-(propan-2-yl)benzene^1^ (1.60 g, 8.34 mmol in dichloromethane (5.0 mL). The mixture was then treated with aluminum trichloride (2.50 g, 18.5 mmol) and the mixture stirred overnight at room temperature then treated with 20% hydrochloric acid in ice and stirred overnight. The mixture was then diluted with additional dichloromethane and washed with 2.0 N NaOH. The organic layer was subsequently dried with sodium sulfate, filtered and concentrated in vacuo. The resulting residue was then purified via MPLC (SiO2, 100% hexanes to 10% ethyl acetate / hexanes (2.10 g, 72% yield). 1H NMR (500 MHz, Chloroform-d) δ 8.17 – 8.12 (m, 2H), 7.82 – 7.76 (m, 3H), 7.62 (dd, J = 8.5, 2.3 Hz, 1H), 6.86 (d, J = 8.5 Hz, 1H), 3.97 (s, 3H), 3.83 (d, J = 6.3 Hz, 2H), 3.37 (hept, J = 7.0 Hz, 1H), 2.17 (hept, J = 6.6 Hz, 1H), 1.25 (d, J = 6.9 Hz, 6H), 1.09 (d, J = 6.7 Hz, 6H).

**Methyl 4-{1-[3-tert-iso-propyl-(2-methylpropoxy)phenyl]-ethenyl}benzoate.** A solution of methyl 4-{1-[3-iso-propyl-4-(2-methylpropoxy)phenyl]-1-hydroxyethyl}benzoate (0.16 g, 0.44 mmol) in toluene (2.0 mL) was stirred at -15 oC under nitrogen atmosphere for 30 minutes. Methylmagnesium iodide (0.20 mL of 3.0 M solution in diethyl ether, 0.57 mmol) was added dropwise over 10 minutes and the reaction mixture stirred at -10^o^C for 30 minutes then warmed to room temperature. Upon completion, the mixture was diluted with ethyl acetate (10 mL) and quenched with aqueous hydrochloric acid (5 mL of 1M solution). The two layers were separated, and the organic layer was dried over anhydrous sodium sulfate, filtered and concentrated in vacuo to provide methyl 4-{1-[4-ethoxy-2-methyl-5-(propan-2-yl)phenyl]-1-hydroxyethyl}benzoate which was used in the next step without further purification. A mixture of crude methyl 4-{1-[4-ethoxy-2-methyl-5-(propan-2-yl)phenyl]-1-hydroxyethyl}benzoate (0.68 mmol) and p-toluene sulfonic acid (0.026 g, 0.14 mmol) in toluene (7.0 mL) in a 25 mL. round-bottom flask, equipped with Dean-Stark trap, was heated to reflux for 24 hours, driving off the water. The mixture was then concentrated in vacuo, diluted with ethyl acetate then washed with water. The organic layer was then dried with sodium sulfate, filtered and concentrated in vacuo. The residue was then purified via MPLC (SiO_2_, 100% hexanes to 10% ethyl acetate / hexanes to provide a solid (0.11 g, 71% yield). 1H NMR (500 MHz, Chloroform-d) δ 8.04 – 7.98 (m, 2H), 7.48 – 7.41 (m, 2H), 7.18 (d, J = 2.4 Hz, 1H), 7.07 (dd, J = 8.4, 2.4 Hz, 1H), 6.78 (d, J = 8.5 Hz, 1H), 5.49 (d, J = 1.1 Hz, 1H), 5.43 (d, J = 1.2 Hz, 1H), 3.94 (s, 3H), 3.76 (d, J = 6.3 Hz, 2H), 3.37 (dp, J = 17.3, 6.9 Hz, 1H), 2.23 – 2.12 (m, 1H), 1.21 (d, J = 6.9 Hz, 6H), 1.07 (d, J = 6.7 Hz, 6H).

**4-{1-[3-Iso-propyl-4-(2-methylpropoxy)phenyl]ethenyl}benzoic acid (MSU-41405).** A mixture of methyl 4-{1-[3-tert-iso-propyl-(2-methylpropoxy)phenyl]-ethenyl}benzoate (0.11 g, 0.32 mmol), potassium hydroxide (0.04 g, 0.63 mmol), methanol (3.0 mL) and water (1.0 mL) was heated to 80^o^C for 6 hrs. The mixture was cooled and acidified with 1.0 N hydrochloric acid and extracted with ethyl acetate (3 times). The organic layers were combined, dried with magnesium sulfate, and the solvent removed in vacuo to provide a solid (0.073 g, 67%). 1H NMR (500 MHz, Chloroform-d) δ 8.10 – 8.04 (m, 2H), 7.49 – 7.43 (m, 2H), 7.18 (d, J = 2.3 Hz, 1H), 7.06 (dd, J = 8.4, 2.4 Hz, 1H), 6.78 (d, J = 8.4 Hz, 1H), 5.50 (d, J = 1.1 Hz, 1H), 5.44 (d, J = 1.1 Hz, 1H), 3.76 (d, J = 6.3 Hz, 2H), 3.34 (hept, J = 6.8 Hz, 1H), 2.14 (dp, J = 13.2, 6.6 Hz, 1H), 1.21 (d, J = 6.9 Hz, 6H), 1.07 (d, J = 6.7 Hz, 6H). HRMS (ES-) (M+H) Calc. 337.1798, Found 337.1818.

**MSU-41407**

**Methyl 4-[2-methyl-5-(propan-2-yl)-4-(propan-2-yloxy)benzoyl]benzoate.** A mixture of thymol (4.00 g, 26.6 mmol) and acetone (100 mL) was treated with 2-iodopropane (13.6 g, 79.9 mmol) and potassium carbonate (4.60 g, 33.33 mmol) and heated to reflux for 5 days. The mixture was cooled, diluted with hexanes, then filtered through a plug of SiO_2_ to provide 4-methyl-1-(propan-2-yl)-2-(propan-2-yloxy)benzene upon concentration, as an oil (5.12 g, 76%), which was used without further purification. A stirred slurry of 4-(methoxycarbonyl)benzoic acid (2.00 g, 11.1 mmol) in dichloromethane (12.0 mL) was treated with oxalyl chloride (1.42 mL, 16.7 mmol) and treated with 1 drop of dimethylformamide. The mixture was stirred for 30 minutes to provide a homogeneous solution, which was concentrated in vacuo. The resulting residue was then re-dissolved in dichloromethane (10.0 mL) solution again concentrated *in vacuo*, this process repeated 3 times. The residue was then again resuspended in dichloromethane (20.0 mL) and treated with a solution of 4-methyl-1-(propan-2-yl)-2-(propan-2-yloxy)benzene (1.91 g, 10.0 mmol) in dichloromethane (5.0 mL). The mixture was then treated with aluminum trichloride (2.96 g, 22.2 mmol), stirred overnight at room temperature and treated with 20% hydrochloric acid in ice, the mixture stirred overnight. It was then diluted with additional dichloromethane and washed with 2.0 N NaOH. The organic layer was subsequently dried with sodium sulfate, filtered and concentrated *in vacuo*. The resulting residue was purified via MPLC (SiO2, 100% hexanes to 10% ethyl acetate / hexanes (2.30 g, 65% yield). 1H NMR (500 MHz, Chloroform-d) δ 8.14 – 8.08 (m, 2H), 7.85 – 7.78 (m, 2H), 7.21 (s, 1H), 6.74 (t, J = 0.8 Hz, 1H), 4.67 (pd, J = 6.1, 0.7 Hz, 1H), 3.94 (s, 3H), 3.25 (hept, J = 6.9 Hz, 1H), 2.41 (d, J = 0.7 Hz, 3H), 1.38 (d, J = 6.1 Hz, 6H), 1.12 (d, J = 6.9 Hz, 6H).

**Methyl 4-{1-[2-methyl-5-(propan-2-yl)-4-(propan-2-yloxy)phenyl]ethenyl}benzoate.** A solution of methyl 4-[2-methyl-5-(propan-2-yl)-4-(propan-2-yloxy)benzoyl]benzoate (0.24 g, 0.68 mmol) in toluene (3.0 mL) was stirred at -15 oC under nitrogen atmosphere for 30 minutes. Methylmagnesium iodide (0.30 mL of 3.0 M solution in diethyl ether, 0.89 mmol) was added dropwise for 10 minutes and the reaction mixture to stirred at -10^o^C for 30 minutes and warmed to room temperature. Upon completion, the reaction was diluted with ethyl acetate (10 mL) and quenched with aqueous hydrochloric acid (5 mL of 1M solution). The two layers were separated and the organic layer was dried over anhydrous sodium sulfate, filtered and concentrated in vacuo into residue methyl 4-{1-hydroxy-1-[2-methyl-5-(propan-2-yl)-4-(propan-2 yloxy)phenyl]ethyl}benzoate which was used in the next step without further purification. This product (0.68 mmol) and p-toluene sulfonic acid (0.026 g, 0.14 mmol) were dissolved in toluene (7.0 mL) in a round-bottom flask equipped with Dean-Stark trap and heated to reflux for 24 hours, driving off the water. The mixture was then concentrated in vacuo, diluted with ethyl acetate then washed with water. The organic layer was then dried with sodium sulfate, filtered and concentrated in vacuo. The residue was then purified via MPLC (SiO_2_, 100% hexanes to 10% ethyl acetate/hexanes to provide a solid (0.13 g, 51% yield). 1H NMR (500 MHz, Chloroform-d) δ 7.99 – 7.91 (m, 2H), 7.38 – 7.29 (m, 2H), 7.02 (s, 1H), 6.66 (s, 1H), 5.80 (d, J = 1.4 Hz, 1H), 5.30 (d, J = 1.5 Hz, 1H), 4.57 (hept, J = 6.1 Hz, 1H), 3.91 (s, 3H), 3.28 (tt, J = 12.0, 6.9 Hz, 1H), 1.96 (s, 3H), 1.38 (m, 6H), 0.92 – 0.82 (m, 6H).

**4-{1-[2-Methyl-5-(propan-2-yl)-4-(propan-2-yloxy)phenyl]ethenyl}benzoic acid** **(MSU-41407).** A mixture of methyl 4-{1-[2-methyl-5-(propan-2-yl)-4-(propan-2-yloxy)phenyl]ethenyl}benzoate (0.11 g, 0.30 mmol), potassium hydroxide (0.03 g, 0.50 mmol), methanol (3.0 mL) and water (1.0 mL) was heated to 80^o^C for 6 hrs. The mixture was cooled and acidified with 1.0 N hydrochloric acid and extracted with ethyl acetate (3 times). The organic layers were combined, dried with magnesium sulfate, filtered and the solvent removed in vacuo to provide a solid (0.075 g, 74%).1H NMR (500 MHz, Chloroform-d) δ 8.01 (d, J = 8.1 Hz, 2H), 7.38 (d, J = 8.1 Hz, 2H), 7.02 (s, 1H), 6.66 (s, 1H), 5.84 – 5.80 (m, 1H), 5.33 (d, J = 1.4 Hz, 1H), 4.57 (hept, J = 6.1 Hz, 1H), 3.29 (p, J = 6.9 Hz, 1H), 2.13 (d, J = 7.3 Hz, 1H), 1.96 (s, 3H), 1.37 (d, J = 6.0 Hz, 6H), 1.26 – 1.17 (m, 6H). HRMS (ES+) (M+H) Calc. 337.1799, Found 337.1800.

**MSU-41406**

**1-iso-butyl-4-methyl-2-(2-methylpropoxy)benzene.** A mixture of 5-methyl-2-(propan-2-yl)phenol (4.00 g, 19.3 mmol) and dimethylformamide (15.0 mL) was treated with isobutyl iodide (4.00 g, 26.6 mmol) and potassium carbonate (4.14 g, 30.0 mmol) and heated to 80^o^C for 3 days. The mixture was cooled and diluted with water. The mixture was then extracted with hexanes (3 times). The organic layers were combined and filtered through a plug of SiO_2_ to provide an oil (1.7 g, 38% yield), which was used without further purification. 1H NMR (500 MHz, Chloroform-d) δ 7.10 (d, J = 7.6 Hz, 1H), 6.74 (dtd, J = 7.7, 1.2, 0.6 Hz, 1H), 6.66 (d, J = 1.6 Hz, 1H), 3.73 (d, J = 6.3 Hz, 2H), 3.32 (hept, J = 6.9 Hz, 1H), 2.33 (d, J = 0.6 Hz, 3H), 2.13 (dp, J = 13.1, 6.6 Hz, 1H), 1.22 (d, J = 6.9 Hz, 6H), 1.06 (d, J = 6.7 Hz, 6H).

**Methyl 4-[5-iso-propyl-2-methyl-4-(2-methylpropoxy)benzoyl]benzoate.** A stirred slurry of 4-(methoxycarbonyl)benzoic acid (2.00 g, 11.10 mmol) in dichloromethane (12.0 mL) was treated with oxalyl chloride (1.42 mL, 16.7 mmol) and treated with 1 drop of dimethylformamide. The mixture was stirred for 30 minutes to provide a homogeneous solution, which was concentrated in vacuo. The resulting residue was then re-dissolved in dichloromethane (10.0 mL) and the solution again concentrated in vacuo, this process repeated 3 times. The residue was then again resuspended in dichloromethane (20.0 mL) and treated with a solution of 1-iso-butyl-4-methyl-2-(2-methylpropoxy)benzene (2.05 g, 10.0 mmol) in dichloromethane (5.0 mL). The mixture was then treated with aluminum trichloride (2.96 g, 22.20 mmol) and the mixture stirred overnight at room temperature. The mixture was then treated with 20% hydrochloric acid in ice and the mixture stirred overnight. The mixture was then diluted with additional dichloromethane and washed with 2.0 N NaOH. The organic layer was subsequently dried with sodium sulfate, filtered and concentrated in vacuo. The resulting residue was then purified via MPLC (SiO_2_, 100% hexanes to 10% ethyl acetate/hexanes (2.29 g, 62% yield). 1H NMR (500 MHz, Chloroform-d) δ 8.14 (d, J = 1.3 Hz, 0H), 8.09 (q, J = 1.4 Hz, 2H), 7.83 – 7.76 (m, 2H), 7.20 (d, J = 1.4 Hz, 1H), 6.72 (s, 1H), 3.95 (s, 3H), 3.80 (dd, J = 6.4, 1.9 Hz, 2H), 3.28 (pd, J = 6.9, 1.7 Hz, 1H), 2.40 (d, J = 1.9 Hz, 3H), 2.15 (dtd, J = 13.2, 6.5, 2.4 Hz, 1H), 1.14 (dd, J = 6.9, 1.9 Hz, 6H), 1.07 (ddd, J = 6.7, 2.4, 1.4 Hz, 6H).

**Methyl 4-{1-[5- iso-propyl-2-methyl-4-(2 methylpropoxy)-phenyl]ethenyl}benzoate.** A solution of methyl 4-[5-iso-propyl-2-methyl-4-(2-methylpropoxy)benzoyl]benzoate (0.30 g, 0.82 mmol) in toluene (3.0 mL) was stirred at -15 ^o^C under nitrogen atmosphere for 30 minutes. Methylmagnesium iodide (0.36 mL of 3M solution in diethyl ether, 1.07 mmol) was added dropwise for 10 minutes and the reaction mixture was continued to stir at -10^o^C for 30 minutes and warmed to room temperature. Upon completion, the reaction was diluted with ethyl acetate (10 mL) and quenched with aqueous hydrochloric acid (5 mL of 1M solution). The two layers were separated, and the organic layer was dried over anhydrous sodium sulfate, filtered and concentrated in vacuo into residue methyl 4-{1-[5-iso-propyl-2-methyl-4-(2-methylpropoxy)phenyl]-1-hydroxyethyl}benzoate which was used in the next step without further purification. The crude methyl 4-{1-[5- iso-propyl -2-methyl-4-(2-methylpropoxy)phenyl]-1-hydroxyethyl}benzoate (0.82 mmol) and p-toluene sulfonic acid (0.07 g, 0.41 mmol) in toluene (7.0 mL) in a 25 mL round-bottom flask, equipped with Dean-Stark trap, was heated to reflux for 24 hours, driving off the water. The mixture was then concentrated in vacuo and diluted with ethyl acetate, washed with water. The organic layer and dried with sodium sulfate, filtered and concentrated in vacuo. The residue was then purified via MPLC (SiO_2_, 100% hexanes to 10% ethyl acetate / hexanes to provide a solid (0.21 g, 68% yield). 1H NMR (500 MHz, Chloroform-d) δ 7.95 (d, J = 8.6 Hz, 2H), 7.35 (d, J = 8.6 Hz, 2H),), 7.03 (s, 1H), 6.64 (s, 1H), 5.81 (s, 1H), 5.29 (d, J = 1.4 Hz, 1H), 4.46 – 4.33 (m, 1H), 3.96 – 3.88 (s, 3H), 3.75 (d, J = 6.2 Hz, 2H), 3.32 (dp, J = 10.1, 6.8 Hz, 1H), 1.97 (s, 3H), 1.23 (d, J = 6.9 Hz, 6H), 1.07 (d, J = 6.7 Hz, 6H).

**4-{1-[5- iso-propyl-2-methyl-4-(2-methylpropoxy)phenyl]-ethenyl}benzoic acid (MSU-41406).** A mixture of methyl 4-{1-[5- iso-propyl-2-methyl-4-(2 methylpropoxy)phenyl]-ethenyl}benzoate (0.07 g, 0.20 mmol), potassium hydroxide (0.03 g, 0.40 mmol), methanol (3.0 mL) and water (1.0 mL) was heated to 80^o^C for 6 hrs. The mixture was cooled and acidified with 1.0 N hydrochloric acid and extracted with ethyl acetate (3 times). The organic layers were combined, dried with magnesium sulfate, and the solvent removed in vacuo to provide a solid (0.065 g, 92%). 1H NMR (500 MHz, Chloroform-d) δ 8.02 (dd, J = 8.5, 2.0 Hz, 2H), 7.41 – 7.36 (m, 2H), 7.04 (s, 1H), 6.65 (s, 1H), 5.84 (d, J = 1.3 Hz, 1H), 5.33 (d, J = 1.2 Hz, 1H), 3.76 (d, J = 6.2 Hz, 2H), 3.34 (p, J = 6.9 Hz, 1H), 2.14 (hept, J = 6.5 Hz, 1H), 1.99 (s, 3H), 1.24 (d, J = 6.9 Hz, 6H), 1.08 (d, J = 6.8 Hz, 6H). HRMS (ES-) (M+H) Calc. 351.1955, Found 351.1961.

**MSU-41408**

**2-ethoxy-4-methyl-1-(propan-2-yl)benzene.** A mixture of thymol (8.00 g, 53.3 mmol) and acetone (200 mL) was treated with bromoethane (19.9 g, 109 mmol) and potassium carbonate (18.4 g, 133 mmol) and heated to reflux for 4 days. The mixture was cooled, diluted with hexanes, then filtered through a plug of SiO2 to up on concentration, provide an oil (8.7 g, 93%), which was used without further purification. 1H NMR (500 MHz, Chloroform-d) δ 7.09 (d, J = 7.7 Hz, 1H), 6.77 – 6.71 (m, 1H), 6.66 (d, J = 1.7 Hz, 1H), 4.03 (q, J = 7.0 Hz, 2H), 3.29 (h, J = 6.9 Hz, 1H), 2.34 – 2.30 (m, 3H), 1.42 (td, J = 7.0, 0.5 Hz, 3H), 1.28 – 1.23 (m, 1H), 1.21 (dd, J = 6.9, 0.7 Hz, 6H).

**Methyl 4-[4-ethoxy-2-methyl-5-(propan-2-yl)benzoyl]benzoate.** A stirred slurry of 4-(methoxycarbonyl)benzoic acid (2.00 g, 11.10 mmol) in dichloromethane (12.0 mL) was treated with oxalyl chloride (1.42 mL, 16.66 mmol), treated with 3 drops of dimethylformamide and stirred for 30 minutes to provide a homogeneous solution, which was concentrated in vacuo. The resulting residue was then re-dissolved in dichloromethane (10.0 mL) and the solution again concentrated in vacuo, this process repeated 3 times. The residue was resuspended in dichloromethane (20.0 mL) and treated with a solution of 2-ethoxy-4-methyl-1-(propan-2-yl)benzene (1.77 g, 10.0 mmol) in dichloromethane (5.0 mL) and treated with aluminum trichloride (2.96 g, 22.2 mmol) and the mixture stirred overnight at room temperature. The mixture was then treated with 20% hydrochloric acid in ice and stirred overnight then diluted with additional dichloromethane and washed with 2.0 N NaOH. The organic layer was subsequently dried with sodium sulfate, filtered and concentrated in vacuo. The resulting residue was then purified via MPLC (SiO_2_, 100% hexanes to 10% ethyl acetate/hexanes (2.41 g, 71% yield). 1H NMR (500 MHz, Chloroform-d) δ 8.12 – 8.08 (m, 2H), 7.84 – 7.77 (m, 2H), 7.20 (s, 1H), 6.73 (s, 1H), 4.11 (q, J = 7.0 Hz, 2H), 3.95 (s, 3H), 3.27 (hept, J = 6.9 Hz, 1H), 2.41 (s, 3H), 1.46 (t, J = 7.0 Hz, 3H), 1.13 (d, J = 6.9 Hz, 6H).

**Methyl 4-{1-[4-ethoxy-2-methyl-5-(propan-2-yl)phenyl]ethenyl}benzoate.** A solution of methyl 4-[4-ethoxy-2-methyl-5-(propan-2-yl)benzoyl]benzoate (0.25 g, 0.77 mmol) in toluene (3.0 mL) was stirred at -15 oC under nitrogen atmosphere for 30 minutes. Methylmagnesium iodide (0.34 mL of 3M solution in diethyl ether, 1.0 mmol) was added dropwise for 10 minutes stirred at -10^o^C for 30 minutes then warmed to room temperature. Upon completion, the reaction was diluted with ethyl acetate (10 mL) and quenched with aqueous hydrochloric acid (5.0 mL of 1.0 M solution). The two layers were separated, and the organic layer dried over anhydrous sodium sulfate, filtered and concentrated in vacuo to provide methyl 4-{1-[4-ethoxy-2-methyl-5-(propan-2-yl)phenyl]-1-hydroxyethyl}benzoate which was used in the next step without further purification. A mixture of crude methyl 4-{1-[4-ethoxy-2-methyl-5-(propan-2-yl)phenyl]-1-hydroxyethyl}benzoate (0.77 mmol) and p-toluene sulfonic acid (0.68 g, 0.39 mmol) in toluene (7.0 mL) in a 25 mL round-bottom flask equipped with Dean-Stark trap was heated to reflux for 24 hours, driving off the water. The mixture was then concentrated in vacuo, diluted with ethyl acetate then washed with water. The organic layer was then dried with sodium sulfate, filtered and concentrated in vacuo. The residue was then purified via MPLC (SiO_2_, 100% hexanes to 10% ethyl acetate / hexanes to provide a solid (0.22 g, 82% yield). 1H NMR (500 MHz, Chloroform-d) δ 7.93 (d, J = 8.3 Hz, 2H), 7.32 (d, J = 8.5 Hz, 2H), 7.00 (s, 1H), 6.63 (s, 1H), 5.79 (d, J = 1.4 Hz, 1H), 5.27 (d, J = 1.5 Hz, 1H), 3.88 (s, 3H), 3.34 – 3.23 (m, 1H), 2.35 (s, 3H), 1.95 (s, 3H), 1.43 (m, 3H), 1.15 (d, J = 7.0 Hz, 6H).

**4-{1-[4-ethoxy-2-methyl-5-(propan-2-yl)phenyl]ethenyl}benzoic acid (MSU-41408).** A mixture of methyl 4-{1-[4-ethoxy-2-methyl-5-(propan-2-yl)phenyl]ethenyl}benzoate (0.20 g, 0.59 mmol), potassium hydroxide (0.06 g, 1.00 mmol), methanol (3.0 mL) and water (1.0 mL) was heated to 80^o^C for 6 hrs. The mixture was cooled and acidified with 1.0 N hydrochloric acid and extracted with ethyl acetate (3 times). The organic layers were combined, dried with magnesium sulfate, and the solvent removed in vacuo to provide a solid (0.15 g, 76%). 1H NMR (500 MHz, Chloroform-d) δ 8.05 – 7.96 (m, 2H), 7.40 – 7.35 (m, 2H), 7.03 (s, 1H), 6.66 (s, 1H), 5.83 (d, J = 1.3 Hz, 1H), 5.32 (d, J = 1.4 Hz, 1H), 4.06 (q, J = 6.9 Hz, 2H), 3.31 (hept, J = 6.9 Hz, 1H), 1.98 (s, 3H), 1.44 (t, J = 6.9 Hz, 3H), 1.22 (d, J = 6.9 Hz, 6H). HRMS (ES-) (M+H) Calc. 323.1642, Found 323.1648.

**MSU-41403**

**2,4-di-tert-butyl-1-ethoxybenzene.** A mixture of 2,4-di-tert-butyl-phenol (4.00 g, 19.3 mmol) and acetone (200 mL) was treated with iodoethane (10.6 g, 96.9 mmol) and potassium carbonate (7.88 g, 57.0 mmol) and heated to reflux for 3 days. The mixture was cooled, diluted with hexanes, then filtered through a plug of SiO_2_, washing with hexanes, provide an oil (4.5 g, 99%), which was used without further purification. 1H NMR (500 MHz, Chloroform-d) δ 7.33 (dd, J = 9.3, 2.5 Hz, 1H), 7.18 (dd, J = 8.4, 2.5 Hz, 1H), 6.80 (d, J = 8.4 Hz, 1H), 4.05 (q, J = 7.0 Hz, 2H), 1.47 (t, J = 7.0 Hz, 3H), 1.42 (s, 9H), 1.32 (s, 9H).

**Methyl 4-(5-tert-butyl-2-ethoxybenzoyl)benzoate.** A stirred slurry of 4-(methoxycarbonyl)benzoic acid (1.00 g, 5.55 mmol) in dichloromethane (10.0 mL) was treated with oxalyl chloride (0.71 mL, 8.81 mmol) and treated with 1 drop of dimethylformamide. The mixture was stirred for 30 minutes to provide a homogeneous solution, which was concentrated in vacuo. The resulting residue was then re-dissolved in dichloromethane (10.0 mL) and the solution again concentrated in vacuo, this process repeated 3 times. The residue was then again resuspended in dichloromethane (20.0 mL) and treated with a solution of 2,4-di-tert-butyl-1-ethoxybenzene (1.16 g, 4.95 mmol) in dichloromethane (5.0 mL). The mixture was then treated with aluminum trichloride and the mixture stirred overnight at room temperature. The mixture was then treated with 20% hydrochloric acid in ice and the mixture stirred overnight. The mixture was then diluted with additional dichloromethane and washed with 2.0 N NaOH. The organic layer was subsequently dried with sodium sulfate, filtered and concentrated in vacuo. The resulting residue was then purified via MPLC (SiO_2_, 100% hexanes to 10% ethyl acetate / hexanes (0.480 g, 29% yield). 1H NMR (500 MHz, Chloroform-d) δ 8.13 – 8.06 (m, 2H), 7.86 – 7.79 (m, 2H), 7.54 – 7.48 (m, 2H), 6.89 (dd, J = 8.2, 0.9 Hz, 1H), 3.96 (s, 3H), 3.90 (q, J = 6.9 Hz, 2H), 1.33 (s, 9H), 0.98 (t, J = 6.9 Hz, 3H).

**4-[1-(5-tert-butyl-2-ethoxyphenyl)ethenyl]benzoic acid (MSU-41403).** A solution of methyl 4-(5-tert-butyl-2-ethoxybenzoyl)benzoate (0.200 g, 0.500 mmol) in dry tetrahydrofuran (2.0 mL), under a nitrogen atmosphere, was transferred via canula to a cooled (0^o^C) solution of triphenylmethylphosphonium bromide (0.450 g, 1.26 mmol) and 2.5 M n-butyllithium (0.48 mL, 1.26 mmol) in dry tetrahydrofuran (5.0 mL). The mixture was allowed to warm to room temperature and stirred overnight, then quenched with 1.0 N hydrochloric acid and extracted with ethyl acetate 3 times. The organic layers were combined, dried with sodium sulfate, filtered, and concentrated in vacuo. The resulting residue was used without further purification. Crude methyl 4-[1-(5-tert-butyl-2-ethoxyphenyl)-1-hydroxyethyl]benzoate (0.078 g, 0.20 mmol), potassium hydroxide, methanol (3.0 mL) and water (1.0 mL) was heated to 80^o^C for 6 hrs. The mixture was cooled and acidified with 1.0 N hydrochloric acid and extracted with ethyl acetate (3 times). The organic layers were combined, dried with magnesium sulfate, and the solvent removed in vacuo, and submitted to MPLC (SiO_2_, hexanes to 20% ethyl acetate / hexanes) to provide a solid (0.048 g, 69%). 1H NMR (500 MHz, Chloroform-d) δ 8.06 – 7.98 (m, 2H), 7.42 – 7.36 (m, 2H), 7.39 – 7.30 (m, 2H), 6.80 (dd, J = 8.2, 0.7 Hz, 1H), 5.73 (d, J = 1.3 Hz, 1H), 5.47 (d, J = 1.3 Hz, 1H), 3.79 (q, J = 7.0 Hz, 2H), 1.34 (s, 9H),, 0.91 (t, J = 6.9 Hz, 3H). HRMS (ES-) (M-H) Calc. 323.1642, Found 323.1669

**MSU-41845**

**1-bromo-2-methyl-4-(2-methylpropoxy)-5-(propan-2-yl)benzene.** 1-(2-methylpropoxy)-2-(propan-2-yl)benzene (1.0 g, 4.8 mmol) was dissolved in acetonitrile (15.0 mL) and treated with n-bromosuccinimide (0.91 g, 5.1 mmol) and the mixture allowed to stir overnight. The mixture was concentrated in vacuo then filtered through a SiO2 plug with hexanes. The solvent was then removed in vacuo to provide an oil (1.2 g, 86%) used without further purification.

**Methyl 4-{[2-methyl-4-(2-methylpropoxy)-5-(propan-2-yl)phenyl]amino}benzoate.** A mixture of 1-bromo-2-methyl-4-(2-methylpropoxy)-5-(propan-2-yl)benzene (0.20 g, 0.71 mmol), methyl 4-aminobenzoate (0.18 g, g, 1.2 mmol), cesium carbonate (0.46 g, 1.4 mmol), (±)-2,2′-Bis(diphenylphosphino)-1,1′-binaphthalene (0.044 g, 0.071 mmol) and palladium acetate (0.016 g, 0.071 mmol) in toluene (3.0 mL) was sealed in a microwave vial and heated by microwave to 110 ^o^C for 18 hrs. The mixture was then diluted into hexanes and Celite added and stirred. After 30 minutes, the mixture was filtered through a pad of Celite and the solvent removed in vacuo. The residue was purified via MPLC (SiO_2_, 100% hexanes gradient to 30% ethyl acetate) to provide a solid (0.17 g, 67%). 1H NMR (500 MHz, Chloroform-d) δ 7.86 (dd, J = 8.9, 2.4 Hz, 2H), 7.06 (s, 1H), 6.72 (s, 1H), 6.61 (s, 1H), 6.62 – 6.57 (m, 1H), 3.86 (s, 3H), 3.75 (d, J = 6.3 Hz, 2H), 3.32 (hept, J = 6.9 Hz, 1H), 2.18 (s, 3H), 2.13 (dq, J = 13.2, 6.6 Hz, 1H), 1.20 (d, J = 6.9 Hz, 6H), 1.08 (d, J = 6.7 Hz, 6H).

**4-{ethyl[2-methyl-4-(2-methylpropoxy)-5-(propan-2-yl)phenyl]amino}benzoic acid (MSU-41845).** A solution of methyl 4-{[2-methyl-4-(2-methylpropoxy)-5-(propan-2-yl)phenyl]amino}benzoate (0.17 g, 0.46 mmol) in dimethylformamide (2.0 mL) was treated with 60%sodium hydride (0.037 g, 0.93 mmol), stirred for 15 minutes, then treated with iodoethane (0.074 mL, 0.93 mmol) and the mixture stirred at room temperature for 18 hours. The mixture was then quenched with an aqueous solution of saturated ammonium chloride and extracted with ethyl acetate. The organic layers were combined, dried with sodium sulfate, filtered and the solvent removed in vacuo. The crude residue was then used in the next step. A mixture of methyl 4-{ethyl[2-methyl-4-(2-methylpropoxy)-5-(propan-2-yl)phenyl]amino}benzoate (0.065 g, 0.17 mmol), potassium hydroxide (0.095 g, 1.7 mmol), methanol (4.0 mL) and water (1.0 mL) was heated to 70^o^C overnight. The mixture was cooled and concentrated in vacuo. The residue was then redissolved in methanol, treated with 4.0 N HCl in dioxane (1.0 mL) and stirred for 20 minutes. The mixture was then filtered to remove solids and the solvent removed in vacuo to provide a solid (0.052, 83%). 1H NMR (500 MHz, DMSO-d6) δ 7.73 – 7.62 (m, 2H), 6.89 (d, J = 10.8 Hz, 2H), 6.39 (d, J = 8.6 Hz, 2H), 3.78 (d, J = 6.2 Hz, 2H), 3.21 (p, J = 6.9 Hz, 1H), 2.06 (dt, J = 13.1, 6.6 Hz, 1H), 1.98 (s, 3H), 1.18 – 1.10 (d, 6H), 1.09 (t, J = 7.0 Hz, 3H), 1.02 (d, J = 6.7 Hz, 6H). HRMS (ES+) (M+H) Calc. 370.2374, Found 370.2409

**MSU-41583**

**Methyl 6-[(5-tert-butyl-2-methylphenyl)amino]pyridine-3-carboxylate.** 5-tert-butyl-2-methylaniline ^2^ (0.15 g, 0.94 mmol), methyl 6-chloropyridine-3-carboxylate (0.17 g, 0.99 mmol), p-toluenesulfonic acid (0.16 g, 0.99 mmol) in dioxane (2 mL) was sealed in a microwave vessel and heated to 115^o^C for 19 hours. The mixture was then diluted with ethyl acetate, washed with 2.0 N sodium hydroxide and the organic layer dried over sodium sulfate, filtered and the solvent removed in vacuo. The residue was then purified by MPLC (SiO_2_, 100% dichloromethane gradient to 2% methanol / dichloromethane) to provide a solid (0.061 g, 23% yield), which was filtered and used without further purification. 1H NMR (500 MHz, Chloroform-d) δ 8.76 (d, J = 2.4 Hz, 1H), 8.09 (dd, J = 9.0, 2.2 Hz, 1H), 7.34 (s, 1H), 7.25 (d, J = 1.6 Hz, 2H), 6.59 – 6.53 (m, 1H), 3.91 (s, 3H), 2.24 (s, 3H), 1.32 (s, 9H).

**Methyl 6-[(5-tert-butyl-2-methylphenyl)(ethyl)amino]pyridine-3-carboxylate.** A solution of methyl 6-[(5-tert-butyl-2-methylphenyl)amino]pyridine-3-carboxylate (0.061 g, 0.20 mmol) in dimethylformamide (3.0 mL) was treated with 60% sodium hydride (0.016 g, 0.41 mmol), stirred for 15 minutes, then treated with iodoethane (0.16 mL, 1.0 mmol) and the mixture stirred at room temperature for 72 hours. The mixture was then quenched with an aqueous solution of saturated ammonium chloride and extracted with ethyl acetate. The organic layers were combined, dried with sodium sulfate, filtered and the solvent removed in vacuo. The residue was then purified by MPLC (SiO_2_, 100% hexanes gradient to 10% ethyl acetate / hexanes) to provide a solid (0.060 g, 92%) which was a mixture of methyl and ethyl esters and moved into the ester hydrolysis without further separation. 1H NMR (500 MHz, Chloroform-d) δ 8.90 – 8.85 (m, 1H), 7.80 (dt, J = 9.1, 3.1 Hz, 1H), 7.42 – 7.33 (m, 1H), 7.36 – 7.27 (m, 2H), 7.14 (d, J = 2.0 Hz, 1H), 4.32 (q, J = 7.1 Hz, 1H), 4.24 (dd, J = 13.8, 6.9 Hz, 2H, ethyl ester), 3.86 (s, 3H, methyl ester), 3.73 (q, J = 7.2 Hz, 1H), 2.09 (s, 3H), 1.37-1.33 (m, 3H, ethyl ester), 1.32 (s, 9H), 1.25 (t, J = 7.1 Hz, 3H).

**6-[(5-tert-butyl-2-methylphenyl)(ethyl)amino]pyridine-3-carboxylic acid (MSU-41583).** A mixture of methyl and ethyl 6-[(5-tert-butyl-2-methylphenyl)(ethyl)amino]pyridine-3-carboxylate (0.053 g, 0.16 mmol), potassium hydroxide (0.091 g, 0.16 mmol), methanol (3.0 mL) and water (1.0 mL) was heated to 70 ^o^C overnight. The mixture was cooled and acidified with 1.0 N hydrochloric acid and extracted with ethyl acetate 3 times. The organic layers were combined, dried with sodium sulfate, filtered and the solvent removed in vacuo to provide a solid (0.025 g, 50% yield). 1H NMR (500 MHz, Methanol-d4) δ 8.74 (d, J = 2.2 Hz, 1H), 7.87 – 7.81 (m, 1H), 7.38 (dd, J = 8.1, 2.1 Hz, 1H), 7.32 (d, J = 8.1 Hz, 1H), 7.18 (d, J = 2.0 Hz, 1H), 5.99 (s, 1H), 4.35 – 4.08 (m, 1H), 3.72 (dd, J = 13.7, 7.0 Hz, 1H), 2.08 (s, 3H), 1.32 (s, 9H), 1.24 (t, J = 7.1 Hz, 3H). HRMS (ES-) (M-H) Calc. 311.1766, Found 311.1755.

**MSU-41567**

**Methyl 4-[(3,5-di-tert-butylphenyl)amino]-3-nitrobenzoate.** A mixture of 3,5-di-t-butylaniline (0.250 g, 1.21 mmol), methyl-(4-iodo-3-nitro)benzoate (0.374 g, 1.22 mmol), cesium carbonate (0.99 g, 3.0 mmol), (2,2′-bis(diphenylphosphino)-1,1′-binaphthyl (0.056 g, 0.090 mmol), tris(dibenzylideneacetone)dipalladium(0) (0.057g, 0.063 mmol) and toluene (6.5 mL) were sealed in a microwave vial and heated to 115^o^C for 19 hrs. The mixture was then cooled and treated with saturated ammonium chloride. The mixture was then extracted with ethyl acetate (3 times) and the organic layers combined, dried with sodium sulfate and the solvent removed in vacuo. The residue was then purified via MPLC (SiO_2_, 100% hexanes to 5% ethyl acetate/hexanes) to provide a solid (0.370 g, 78% yield). 1H NMR (500 MHz, Chloroform-d) δ 9.83 (s, 1H), 8.93 (d, J = 2.1 Hz, 1H), 7.99 – 7.92 (m, 1H), 7.36 (t, J = 1.9 Hz, 1H), 7.16 (d, J = 9.1 Hz, 1H), 7.13 – 7.06 (m, 2H), 3.91 (s, 3H), 1.34 (s, 18H).

**1-(3,5-di-tert-butylphenyl)-2-(trifluoromethyl)-1H-1,3-benzodiazole-5-carboxylic acid (MSU-41567).** A mixture of methyl 4-[(3,5-di-tert-butylphenyl)amino]-3-nitrobenzoate (0.070 g, 0.18 mmol) and zinc dust (0.47 g, 7.2 mmol) in acetic acid (3.0 mL) was stirred overnight and room temperature. The mixture was then diluted with ethyl acetate and filtered, the solids being washed with ethyl acetate. The organics were combined, dried with sodium sulfate, filtered and concentrated in vacuo to provide a solid which was used without further purification being dissolved trifluoroacetic anhydride (1.0 mL) and heated to 60^o^C for 18 hrs. The mixture was then concentrated in vacuo, diluted with hexanes and then washed with a saturated solution of sodium bicarbonate. The organic layer was then dried with sodium sulfate, filtered and concentrated in vacuo. The resulting residue was then filtered (SiO_2_, 100% hexanes to 5% ethyl acetate / hexanes) and the solvent removed in vacuo to provide a thick oil (0.050 g, 64% crude yield). A mixture of crude methyl 1-(3,5-di-tert-butylphenyl)-2-(trifluoromethyl)-1H-1,3-benzodiazole-5-carboxylate (0.050 g, 0.12 mmol), potassium hydroxide (0.087 g, 1.2 mmol), methanol (4.0 mL) and water (1.0 mL) was heated to 80 ^o^C for overnight. The mixture was cooled and acidified with 2.0 N hydrochloric acid and extracted with ethyl acetate (3 times). The organic layers were combined, dried with sodium sulfate, filtered and the solvent removed in vacuo to provide a solid (0.027 g, 55% yield). 1H NMR (500 MHz, Chloroform-d) δ 8.77 (dd, J = 1.5, 0.7 Hz, 1H), 8.17 (dd, J = 8.7, 1.5 Hz, 1H), 7.64 (t, J = 1.7 Hz, 1H), 7.35 – 7.23 (m, 3H), 1.38 (s, 18H). HRMS (ES+) (M+H) Calc. 419.1944, Found 419.1654.

**MSU-41402**

**Methyl 4-[(3,5-di-tert-butylphenyl)amino]benzoate.** A mixture of 3,5-di-t-butyl-bromobenzene ((0.15 g, 0.55 mmol), methyl 4-aminobenzoate (0.17 g, 1.1 mmol), cesium carbonate (0.36 g, 1.1 mmol), methyl 4-aminobenzoate (0.017 g, 0.028 mmol) and palladium acetate (0.006 g, 0.03 mmol) in toluene (6.0 mL) was sealed in a microwave vial and heated by microwave to 115^o^C for 13 hrs. The mixture was then diluted into hexanes and Celite added and stirred. After 30 minutes, the mixture was filtered through a pad of Celite and the solvent removed in vacuo. The residue was purified via MPLC (SiO_2_, 100% hexanes gradient to 20% ethyl acetate) to provide a solid (0.088 g, 47%). 1H NMR (500 MHz, Chloroform-d) δ 7.91 (d, J = 8.2 Hz, 2H), 7.15 (s, 1H), 7.03 (s, 2H), 6.96 (s, 2H), 3.87 (s, 3H), 1.32 (s, 18H).

**Methyl 4-[(3,5-di-tert-butylphenyl)(ethyl)amino]benzoate.** A solution of methyl 4-[(3,5-di-tert-butylphenyl)amino]benzoate (0.085 g, 0.25 mmol) in dimethylformamide (1.5 mL) was treated with 60%sodium hydride (0.012 g, 0.27 mmol), stirred for 15 minutes, then treated with iodoethane (0.040 mL, 0.50 mmol) and the mixture stirred at room temperature for 1 hour. The mixture was then quenched with an aqueous solution of saturated ammonium chloride and extracted with ethyl acetate. The organic layers were combined, dried with sodium sulfate, filtered and the solvent removed in vacuo. The residue was then purified by MPLC (SiO_2_, 100% hexanes gradient to 5% ethylacetate / hexanes) to provide a solid (0.070 g, 77%). 1H NMR (500 MHz, Chloroform-d) δ 7.85 – 7.79 (m, 2H), 7.31 (t, J = 1.8 Hz, 1H), 7.02 (d, J = 1.8 Hz, 2H), 6.66 – 6.61 (m, 2H), 3.84 (s, 3H), 3.79 (q, J = 7.1 Hz, 2H), 1.31 (s, 18H), 1.26 (t, J = 7.1 Hz, 3H).

**4-[(3,5-di-tert-butylphenyl)(ethyl)amino]benzoic acid (MSU-41402).** A mixture of (methyl 4-[(3,5-di-tert-butylphenyl)(ethyl)amino]benzoate (0.070 g, 0.19 mmol), potassium hydroxide (0.11 g, 1.9 mmol), methanol (4.0 mL) and water (1.0 mL) was heated to 80^o^C for overnight. The mixture was cooled and acidified with 1.0 N hydrochloric acid and extracted with ethyl acetate (3 times). The organic layers were combined, dried with sodium sulfate, filtered and the solvent removed in vacuo to provide a solid (0.064 g, 96% yield). 1H NMR (500 MHz, Chloroform-d) δ 7.91 – 7.84 (m, 2H), 7.34 (t, J = 1.8 Hz, 1H), 7.27 (s, 1H), 7.03 (d, J = 1.8 Hz, 2H), 6.97 (s, 1H), 6.67 – 6.60 (m, 2H), 3.80 (q, J = 7.1 Hz, 2H), 1.33 (s, 18H), 1.28 (t, J = 7.1 Hz, 3H). HRMS (ES-) (M-H) Calc. 352.2276, Found 351.352.2281.

**MSU-41564**

**Methyl 6-[(3,5-di-tert-butylphenyl)amino]pyridine-3-carboxylate.** A mixture of 3,5-di-t-butylaniline (0.15 g, 0.73 mmol), methyl 6-chloropyridine-3-carboxylate (0.125 g, 0.73 mmol), p-toluene sulfonic acid (0.14 g, 0.73 mmol) in dioxane (2.0 mL) was sealed in a microwave vessel and heated to 111^o^C for 14 hours. The mixture was then diluted with ethyl acetate, washed with 2.0 N sodium hydroxide and the organic layer dried over sodium sulfate, filtered and the solvent removed in vacuo. The residue was then purified by MPLC (SiO_2_, 100% dichloromethane gradient to 5% isopropanol / dichloromethane) to provide a solid (0.17 g, 69% yield). 1H NMR (500 MHz, Chloroform-d) δ 8.83 (d, J = 2.4 Hz, 1H), 8.05 (dt, J = 8.9, 2.4 Hz, 1H), 7.24 (p, J = 1.7 Hz, 1H), 7.17 – 7.14 (m, 2H), 6.93 (s, 1H), 6.82 (dd, J = 9.0, 2.2 Hz, 1H), 3.89 (s, 3H), 1.34 (s, 18H).

**Methyl 6-[(3,5-di-tert-butylphenyl)(ethyl)amino]pyridine-3-carboxylate.** A solution of methyl 6-[(3,5-di-tert-butylphenyl)amino]pyridine-3-carboxylate (0.170 g, 0.25 mmol) in dimethylformamide (3.0 mL) was treated with 60% sodium hydride (0.022 g, 0.55 mmol), stirred for 15 minutes, then treated with iodoethane (0.080 mL, 1.0 mmol) and the mixture stirred at room temperature for 1.5 hour. The mixture was then quenched with an aqueous solution of saturated ammonium chloride and extracted with ethyl acetate. The organic layers were combined, dried with sodium sulfate, filtered and the solvent removed in vacuo. The residue was then purified by MPLC (SiO_2_, 100% hexanes gradient to 5% ethyl acetate/hexanes) to provide a solid (0.132 g, 72%). 1H NMR (500 MHz, Chloroform-d) δ 8.86 (dd, J = 2.4, 0.8 Hz, 1H), 7.78 (dd, J = 9.1, 2.4 Hz, 1H), 7.39 (t, J = 1.8 Hz, 1H), 7.04 (d, J = 1.8 Hz, 2H), 6.19 (dd, J = 9.1, 0.7 Hz, 1H), 4.05 (q, J = 7.0 Hz, 2H), 3.86 (s, 3H), 1.33 (s, 18H), 1.25 (t, J = 7.0 Hz, 3H).

**6-[(3,5-di-tert-butylphenyl)(ethyl)amino]pyridine-3-carboxylic acid (MSU-41564).** A mixture of methyl 6-[(3,5-di-tert-butylphenyl)(ethyl)amino]pyridine-3-carboxylate (0.130 g, 0.13 g, .35 mmol), potassium hydroxide (0.20 g, 1.9 mmol), methanol (2.0 mL) and water (2.0 mL) was heated to 80^o^C overnight. The mixture was cooled and acidified with 1.0 N hydrochloric acid and extracted with dichloromethane (3 times). The organic layers were combined, dried with sodium sulfate, filtered and the solvent removed in vacuo to provide a solid (0.108 g, 87% yield). 1H NMR (500 MHz, Chloroform-d) δ 8.94 – 8.90 (m, 1H), 7.81 (dd, J = 9.1, 2.3 Hz, 1H), 7.39 (t, J = 1.8 Hz, 1H), 7.04 (d, J = 1.8 Hz, 2H), 6.18 (dd, J = 9.1, 0.8 Hz, 1H), 4.06 (q, J = 7.0 Hz, 2H), 1.33 (s, 18H), 1.25 (t, J = 7.0 Hz, 3H). HRMS (ES-) (M-H) Calc. 353.2224, Found 353.2263.

**MSU-42185**

**6-[(3,5-di-tert-butylphenyl)(propan-2-yl)amino]pyridine-3-carboxylic acid.** A solution of methyl 2-[(3,5-di-tert-butylphenyl)amino]pyridine-5-carboxylate (0.13 g, 0.37 mmol) in dimethylformamide (5.0 mL) was treated with 60% sodium hydride (0.15 g, 3.6 mmol), stirred for 15 minutes, treated with 2-iodopropropane (0.62 g, 3.6 mmol) and the mixture stirred at 50^o^C for 14 hours. The mixture was then quenched with an aqueous solution of saturated ammonium chloride and extracted with ethyl acetate. The organic layers were combined, dried with sodium sulfate, filtered and the solvent removed in vacuo. The residue was then purified by MPLC (SiO_2_, 100% dichloromethane gradient to 5% methanol/dichloromethane) to provide the acid as an oil (0.054 g, 40%). 1H NMR (500 MHz, DMSO-d6) δ 8.65 (dd, J = 2.4, 0.8 Hz, 1H), 7.72 (dd, J = 9.1, 2.4 Hz, 1H), 7.43 (t, J = 1.8 Hz, 1H), 6.96 (d, J = 1.8 Hz, 2H), 5.84 (dd, J = 9.0, 0.8 Hz, 1H), 5.29 (hept, J = 6.7 Hz, 1H), 1.28 (s, 18H), 1.04 (d, J = 6.7 Hz, 6H). HRMS (ES-) (M-H) Calc. 367.2386, Found 367.2386.

**MSU-41842**

**6-[(5-tert-butyl-2-methylphenyl)(propyl)amino]pyridine-3-carboxylic acid.** A solution of methyl 6-[(5-tert-butyl-2-methylphenyl)amino]pyridine-3-carboxylate (0.066 g, 0.22 mmol) in dimethylformamide (2.0 mL) was treated with 60% sodium hydride (0.088 g, 2.2 mmol), stirred for 15 minutes, treated with 1-iodopropane (0.37 g, 2.2 mmol) and the mixture stirred at 60^o^C for 4 days. The mixture was then quenched with an aqueous solution of saturated ammonium chloride and extracted 3 times with ethyl acetate. The organic layers were combined, dried with sodium sulfate, filtered and the solvent removed *in vacuo*. The residue was then dissolved in methanol (5.0 mL) and treated with water (2.0 mL) and potassium hydroxide (0.25 g, 4.4 mmol). The mixture was heated to reflux 24 hours. The mixture was cooled, treated with saturated ammonium chloride solution, extracted 3 times with ethyl acetate, the organic layers combined, dried with sodium sulfate, filtered, and the solvent removed in vacuo. The residue was then purified by MPLC (SiO_2_, 100% dichloromethane gradient to 5% methanol/dichloromethane) to provide a solid (0.0.40 g, 56%). 1H NMR (500 MHz, DMSO-d6) δ 12.44 (s, 1H), 8.68 – 8.64 (m, 1H), 7.78 (dd, J = 8.9, 2.4 Hz, 1H), 7.36 – 7.28 (m, 2H), 7.16 (d, J = 1.9 Hz, 1H), 5.89 (s, 1H), 4.02 (s, 1H), 3.57 (s, 1H), 1.99 (s, 3H), 1.59 (h, J = 7.5 Hz, 2H), 1.26 (s, 9H), 0.87 (t, J = 7.4 Hz, 3H). HRMS (ES-) (M-H) Calc. 367.2381, Found 367.2395.

**MSU-41843**

**Methyl 6-[(cyclopropylmethyl)(3,5-di-tert-butylphenyl)amino]-pyridine-3-carboxylate.** A solution of methyl 2-[(3,5-di-tert-butylphenyl)amino]pyridine-5-carboxylate (0.150 g, 0.44 mmol) in dimethylformamide (2.0 mL) was treated with 60% sodium hydride (0.071 g, 1.8 mmol), at 0^o^C and stirred for 15 minutes then treated with 1-bromomethylcyclopropane (0.33 mL, 2.4 mmol) and stirred at room temperature overnight The mixture was then quenched with water and extracted with ethyl acetate (3 times). The organic layers were combined, dried with sodium sulfate, filtered and the solvent removed in vacuo. The residue was then purified by MPLC (SiO_2_, 100% dichloromethane gradient to 10% methanol/dichloromethane) to provide a solid (0.054 g, 30%). 1H NMR (500 MHz, DMSO-d6) δ 12.45 (s, 1H), 8.66 (dd, J = 2.4, 0.8 Hz, 1H), 7.78 (dd, J = 9.0, 2.3 Hz, 1H), 7.39 (t, J = 1.8 Hz, 1H), 7.12 (d, J = 1.8 Hz, 2H), 6.14 (dd, J = 8.9, 0.8 Hz, 1H), 3.81 (d, J = 6.9 Hz, 2H), 1.29 (s, 18H), 1.13 – 1.04 (m, 1H), 0.43 – 0.35 (m, 2H), 0.13 – 0.06 (m, 2H).

**6-[(cyclopropylmethyl)(3,5-di-tert-butylphenyl)amino]pyridine-3-carboxylic acid (MSU-41843).** A mixture of methyl 6-[(cyclopropylmethyl)(3,5-di-tert-butylphenyl)amino]-pyridine-3-carboxylate (0.053 g, 0.16 mmol), potassium hydroxide (0.091 g, 0.16 mmol), methanol (3.0 mL) and water (1.0 mL) was heated to 70 ^o^C overnight. The mixture was cooled and acidified with 1.0 N hydrochloric acid and extracted with ethyl acetate (3 times). The organic layers were combined, dried with sodium sulfate, filtered and the solvent removed in vacuo to provide a solid (0.055 g, 42% yield). 1H NMR (500 MHz, DMSO-d6) δ 8.61 (d, J = 2.3 Hz, 1H), 7.83 (d, J = 8.8 Hz, 1H), 7.40 (q, J = 2.1 Hz, 1H), 7.13 – 7.08 (m, 2H), 6.24 (s, 1H), 3.94 – 3.87 (m, 2H), 1.59 (h, J = 7.4 Hz, 2H), 1.29 (s, 18H), 0.87 (t, J = 7.4 Hz, 3H). HRMS (ES-) (M-H) Calc. 379.2390, Found 379.2390.

**MSU-42012**

**6-[(3,5-di-tert-butylphenyl)(butyl)amino]pyridine-3-carboxylic acid.** A solution of methyl 2-[(3,5-di-tert-butylphenyl)amino]pyridine-5-carboxylate (0.12 g, 0.34 mmol) in dimethylformamide (5.0 mL) was treated with 60% sodium hydride (0.068 g, 1.7 mmol), stirred for 15 minutes, then treated with 1-iodobutane (0.31 mL, 1.7 mmol) and the mixture stirred at room temperature for 18 hours. The mixture was then quenched with an aqueous solution of saturated ammonium chloride and extracted with ethyl acetate. The organic layers were combined, dried with sodium sulfate, filtered and the solvent removed in vacuo. The residue was then purified by MPLC (SiO_2_, 100% hexanes gradient to 25% ethyl acetate/hexanes) to provide carboxylic acid as a solid (0.064 g, 40%). 1H NMR (500 MHz, DMSO-d6) δ 8.61 (d, J = 2.4 Hz, 1H), 7.82 – 7.77 (m, 1H), 7.40 – 7.33 (m, 1H), 7.13 – 7.05 (m, 2H), 6.22 (s, 1H), 3.96 – 3.90 (m, 2H), 1.53 (tt, J = 7.8, 6.3 Hz, 2H), 1.28 (s, 18H), 1.22 (d, J = 10.8 Hz, 2H), 0.91 – 0.80 (m, 3H). HRMS (ES-) (M-H) Calc. 381.2537, Found 381.2552.

**MSU-42011**

**6-[(3,5-di-tert-butylphenyl)(2-methylpropyl)amino]pyridine-3-carboxylic acid.** A solution of methyl 6-[(3,5-ditertbutylphenyl)(2-methylphenyl)amino]pyridine-3-carboxylate (2.40 g, 7.06 mmol) in dimethylformamide (71.0 mL) was treated with 60% sodium hydride (2.83 g, 70.6 mmol), stirred for 15 minutes, and treated with 1-iodo-2-methylpropane (13.0 g, 70.6 mmol) and the mixture stirred at 40 ^o^C overnight.The mixture was then again treated with sodium hydride (1.42 g, 35.3 mmol) and 1-iodo-2-methylpropane (6.50 g, 35.3 mmol) and continue to stir at 40^o^C overnight. The mixture was then quenched with an aqueous solution of saturated ammonium chloride and extracted 3 times with ethyl acetate. The organic layers were combined, dried with sodium sulfate, filtered and the solvent removed *in vacuo*. The residue was then dissolved in methanol (200.0 mL) and treated with water (50.0 mL) and potassium hydroxide (20.0 g, 353.0 mmol). The mixture was heated to reflux 24 hours. The mixture was cooled, treated with saturated ammonium chloride solution, extracted 3 times with ethyl acetate, the organic layers combined, dried with sodium sulfate, filtered, and the solvent removed *in vacuo*. The residue was then purified by MPLC (SiO_2_, hexanes/dichloromethane (1:1) to 5% methanol in hexanes/dichloromethane (1:1)) to provide a solid (2.40 g, 89% yield). ^1^H NMR (500 MHz, Methanol-*d*_4_) δ 8.73 (dd, *J* = 2.4, 0.8 Hz, 1H), 7.82 (dd, *J* = 9.1, 2.4 Hz, 1H), 7.46 (t, *J* = 1.7 Hz, 1H), 7.11 (d, *J* = 1.8 Hz, 2H), 6.22 (dd, *J* = 9.1, 0.8 Hz, 1H), 3.88 (d, *J* = 7.3 Hz, 2H), 2.04 – 1.94 (m, 1H), 1.34 (s, 18H), 0.96 (d, *J* = 6.7 Hz, 6H). HRMS (ES-) (M-H) Calc. 381.2537, Found 381.2549.

**MSU-41582**

**Methyl 2-[(3,5-di-tert-butylphenyl)amino]pyrimidine-5-carboxylic acid.** A mixture of 3,5-di-t-butylaniline (0.16 g, 0.80 mmol), methyl 2-chloropyrimidine-5-carboxylate (0.14 g, 0.80 mmol), p-toluene sulfonic acid (0.15 g, 0.80 mmol) in dioxane (2 mL) was sealed in a microwave vessel and heated to 111^o^C for 15 hours. The mixture was then diluted with ethyl acetate, washed with 2.0 N sodium hydroxide and the organic layer dried over sodium sulfate, filtered and the solvent removed in vacuo. The residue was then purified by MPLC (SiO_2_, 100% dichloromethane gradient to %5 isopropanol/ dichloromethane) to provide a solid (0.17 g, 69% yield), which was filtered and used without further purification. 1H NMR (500 MHz, DMSO-d6) δ 9.40 (s, 1H), 8.73 (s, 2H), 7.64 (s, 2H), 6.99 (s, 1H), 1.28 (s, 18H).

**Methyl 2-[(3,5-di-tert-butylphenyl)(ethyl)amino]pyrimidine-5-carboxylic acid.** A solution of methyl 2-[(3,5-di-tert-butylphenyl)amino]pyrimidine-5-carboxylate (0.11 g, 0.33 mmol) in dimethylformamide (2.0 mL) was treated with 60% sodium hydride (0.040 g, 0.99 mmol), stirred for 15 minutes, then treated with iodoethane (0.13 mL, 1.7 mmol) and the mixture heated to 50^o^C. The mixture was then quenched with water and extracted with ethyl acetate (3 times). The organic layers were combined, dried with sodium sulfate, filtered and the solvent removed *in vacuo*. The residue was then purified by MPLC (SiO_2_, 100% dichloromethane gradient to 10% methanol / dichloromethane) to provide a solid (0.048 g, 39%). 1H NMR (500 MHz, DMSO-d6) δ 8.61 (s, 2H), 7.27 (t, J = 1.7 Hz, 1H), 7.02 (d, J = 1.8 Hz, 2H), 3.94 (q, J = 7.0 Hz, 2H), 1.28 (s, 18H), 1.13 (t, J = 7.0 Hz, 4H).

**2-[(3,5-di-tert-butylphenyl)(ethyl)amino]pyrimidine-5-carboxylic acid (MSU-41582).** A mixture of methyl 2-[(3,5-di-tert-butylphenyl)(ethyl)amino]pyrimidine-5-carboxylate (0.048 g, 0.13 mmol), potassium hydroxide (0.072 g, 1.3 mmol), methanol (4.0 mL) and water (1.0 mL) was heated to 70^o^C overnight. The mixture was cooled and acidified with 1.0 N hydrochloric acid and extracted with dichloromethane (3 times). The organic layers were combined, dried with sodium sulfate, filtered and the solvent removed in vacuo to provide a solid (0.028 g, 61% yield). 1H NMR (500 MHz, DMSO-d6) δ 12.84 (s, 1H), 8.73 (s, 2H), 7.33 (t, J = 1.7 Hz, 1H), 7.05 (d, J = 1.7 Hz, 2H), 3.97 (q, J = 7.0 Hz, 2H), 1.28 (s, 18H), 1.15 (t, J = 7.0 Hz, 4H). HRMS (ES-) (M-H) Calc. 354.2177, Found 354.2193

**MSU-42165**

**(2-[(3,5-di-tert-butylphenyl)(2-methylpropyl)amino]pyrimidine-5-carboxylic acid.** A solution of methyl 2-[(3,5-di-tert-butylphenyl)amino]pyrimidine-5-carboxylate (0.13 g, 0.37 mmol) in dimethylformamide (3.0 mL) was treated with 60% sodium hydride (0.073 g, 1.8 mmol), stirred for 15 minutes, treated with 1-bromo-2-methylpropane (0.34 mL, 1.8 mmol) and the mixture stirred at 50^o^C for 12 hours. The mixture was then quenched with an aqueous solution of saturated ammonium chloride and extracted with ethyl acetate. The organic layers were combined, dried with sodium sulfate, filtered and the solvent removed in vacuo. The residue was then purified by MPLC (SiO_2_, 100% dichloromethane gradient to 5% methanol / dichloromethane) to provide a solid (0.130 g, 92%). 1H NMR (500 MHz, DMSO-d6) δ 12.82 (s, 1H), 8.71 (s, 2H), 7.30 (t, J = 1.8 Hz, 1H), 7.08 (d, J = 1.7 Hz, 2H), 3.88 (d, J = 7.4 Hz, 2H), 1.87 (hept, J = 6.9 Hz, 1H), 1.27 (s, 18H), 0.86 (d, J = 6.7 Hz, 6H).HRMS (ES+) (M+H) Calc. 384.2644, Found 384.2694.

**MSU-42166**

**2-[(cyclopropylmethyl)(3,5-di-tert-butylphenyl)amino]pyrimidine-5-carboxylic acid.** A solution of methyl 2-[(3,5-di-tert-butylphenyl)amino]pyrimidine-5-carboxylate (0.13 g, 0.37 mmol) in dimethylformamide (5.0 mL) was treated with 60% sodium hydride (0.073 g, 1.8 mmol), stirred for 15 minutes, treated with (bromomethyl)cyclopropane (0.25 g, 1.8 mmol) and the mixture stirred at 50^o^C for 48 hours. The mixture was then quenched with an aqueous solution of saturated ammonium chloride and extracted with ethyl acetate. The organic layers were combined, dried with sodium sulfate, filtered and the solvent removed in vacuo. The residue was then purified by MPLC (SiO_2_, 100% dichloromethane gradient to 5% methanol / dichloromethane) to provide an oil (0.039 g, 28%). 1H NMR (500 MHz, Methanol-d4) δ 8.78 (s, 2H), 7.46 (t, J = 1.8 Hz, 1H), 7.13 (d, J = 1.7 Hz, 2H), 3.89 (d, J = 7.0 Hz, 2H), 1.35 (s, 18H), 1.31 – 1.27 (m, 1H), 0.52 – 0.42 (m, 2H), 0.15 (dt, J = 6.0, 4.5 Hz, 2H). HRMS (ES+) (M+H) Calc. 382.2494, Found 382.2530.

**MSU-41844**

**Methyl 6-{[3,5-bis(trifluoromethyl)phenyl]amino}pyridine-3-carboxylate.** A mixture of 3,5-bis(trifluoromethyl)aniline (0.30 g, 1.3 mmol), methyl 2-chloropyridine-5-carboxylate (0.20 g, 1.2 mmol), p-toluenesulfonic acid (0.22 g, 0.22 mmol) in dioxane (2.5 mL) was sealed in a microwave vessel and heated to 115^o^C for 14 hours. The mixture was then diluted with ethyl acetate, washed with 2.0 N sodium hydroxide and the organic layer dried over sodium sulfate, filtered and the solvent removed in vacuo. The residue was then purified by MPLC (SiO_2_, 100% hexanes gradient to 20% ethyl acetate/hexanes) to provide a solid (0.40 g, 93% yield).1H NMR (500 MHz, DMSO-d6) δ 8.79 (ddd, J = 12.7, 2.3, 0.8 Hz, 1H), 8.42 (s, 2H), 8.06 – 8.03 (m, 1H), 7.79 (s, 1H) 6.90 (d, J = 9.0, 1H).

**6-{[3,5-bis(trifluoromethyl)phenyl](ethyl)amino}pyridine-3-carboxylic acid (MSU-41844).** A solution of methyl 6-{[3,5-bis(trifluoromethyl)phenyl]amino}pyridine-3-carboxylate (0.10 g, 0.27 mmol) in dimethylformamide (3.0 mL) was treated with 60% sodium hydride (0.033 g, 0.82 mmol), stirred for 5 minutes, treated with iodoethane (0.11 mL, 1.3 mmol), and stirred at room temperature for 15 hours. The reaction was then quenched with an aqueous solution of saturated ammonium chloride and extracted with ethyl acetate (3 times). The organic layers were combined, dried with sodium sulfate, filtered and the solvent removed in vacuo. The residue was then purified by MPLC (SiO_2_, 100% hexanes gradient to 10% ethyl acetate/hexanes) to provide a solid (0.063 g, 60%). A mixture of methyl 6-{[3,5-bis(trifluoromethyl)phenyl](ethyl)amino}pyridine-3-carboxylate (0.060 g, 0.15 mmol), potassium hydroxide (0.082 g, 1.58 mmol), methanol (4.0 mL) and water (1.0 mL) was heated to 70^o^C overnight. The mixture was cooled and concentrated in vacuo. The residue was then redissolved in methanol and treated with 4.0 N HCl in dioxane (1.0 mL) and stirred for 20 minutes. The mixture was then filtered to remove solids and the solvent removed in vacuo to provide a solid (0.050, 83%). 1H NMR (500 MHz, DMSO-d6) δ 8.66 (ddd, J = 12.7, 2.3, 0.8 Hz, 1H), 8.08 – 8.03 (m, 2H), 8.03 – 7.94 (m, 2H), 6.69 (dt, J = 9.0, 1.1 Hz, 1H), 4.06 (q, J = 7.1 Hz, 2H), 1.15 (t, J = 7.0 Hz, 3H). HRMS (ES-) (M-H) Calc. 377.0756, Found 377.0747.

**MSU-41565**

**2-bromo-4,6-di-tert-butylphenol.** A solution of thymol (10.0 g, 48 mmol) in acetonitrile (150 mL) was treated with bromosuccinimide (9.1 g, 51 mmol) and allowed to stir at room temperature overnight. The mixture was concentrated in vacuo and dissolved in dichloromethane. The mixture was then filtered through a plug of SiO2 using hexanes as the eluent. The solvent was then removed in vacuo to provide an oil (13.3 g, 96.3%) that was used without further purification. 1H NMR (500 MHz, Chloroform-d) δ 7.41 (dd, J = 2.4, 1.0 Hz, 1H), 7.29 (dd, J = 2.5, 1.0 Hz, 1H), 1.41 (d, J = 1.1 Hz, 9H), 1.30 (d, J = 1.0 Hz, 9H).

**1-bromo-3,5-di-tert-butyl-2-ethoxybenzene.** A mixture of 2-bromo-4,6-di-tert-butylphenol (3.5 g, 12.3 mmol), iodoethane (3.9 mL, 49 mmol), potassium carbonate (8.5 g, 61 mmol) in acetone (100.0 mL) was heated to reflux for 4 days. The mixture was cooled, concentrated in vacuo and filtered through a plug of SiO2 using hexanes as an eluent. The solvent was then removed in vacuo to provide an oil (3.7 g, 96%) used without further purification. 1H NMR (500 MHz, Chloroform-d) δ 7.40 (d, J = 2.4 Hz, 1H), 7.29 (d, J = 2.5 Hz, 1H), 4.09 (q, J = 7.0 Hz, 2H), 1.46 (t, J = 7.0 Hz, 3H), 1.40 (s, 9H), 1.29 (s, 9H).

**Methyl 4-[(3,5-di-tert-butyl-2-ethoxyphenyl)amino]benzoate.** A mixture of 1-bromo-3,5-di-tert-butyl-2-ethoxybenzene (0.31 g, 1.0 mmol), methyl 4-aminobenzoate (0.23 g, 1.5 mmol), potassium phosphate (0.42 g, 2.0 mmol), 2-dicyclohexylphosphino-2′-(N,N-dimethylamino)biphenyl (0.12 g, 0.30 mmol) and tris(dibenzylideneacetone)dipalladium(0) (0.18 g, 0.20 mmol) in toluene (6.0 mL) was sealed in a microwave vial and heated by microwave to 100^o^C for 36 hrs. The mixture was then diluted into hexanes and Celite added and stirred. After 30 minutes, the mixture was filtered through a pad of Celite and the solvent removed in vacuo. The residue was purified via MPLC (SiO_2_, 100% hexanes gradient to 20% dichloromethane) to provide a solid (0.150 g, 39%).1H NMR (500 MHz, Chloroform-d) δ 7.96 – 7.89 (m, 2H), 7.09 (d, J = 2.4 Hz, 1H), 6.98 – 6.91 (m, 2H), 3.87 (s, 3H), 3.87 – 3.79 (m, 2H), 1.41 (s, 9H), 1.34 – 1.30 (m, 3H), 1.29 (s, 9H).

**Methyl 4-[(3,5-di-tert-butyl-2-ethoxyphenyl)(ethyl)amino]benzoate.** A solution of methyl 4-[(3,5-di-tert-butyl-2-ethoxyphenyl)amino]benzoate (0.15 g, 0.38 mmol) in dimethylformamide (3.0 mL) was treated with 60% sodium hydride (0.018 g, 0.45 mmol), stirred for 15 minutes, then treated with iodoethane (0.060 mL, 0.76 mmol) and the mixture heated to 50^o^C. The mixture was then quenched with water and extracted with ethyl acetate (3 times). The organic layers were combined, dried with sodium sulfate, filtered and the solvent removed in vacuo. The residue was then purified by MPLC (SiO_2_, 100% hexanes gradient to 10% ethyl acetate/hexanes) to provide a solid (0.064 g, 40%). 1H NMR (500 MHz, Chloroform-d) δ 7.89 – 7.82 (m, 2H), 7.28 (d, J = 2.4 Hz, 1H), 6.98 (d, J = 2.5 Hz, 1H), 6.71 – 6.65 (m, 2H), 3.99 – 3.88 (m, 1H), 3.86 (s, 3H), 3.79 (s, 1H), 3.63 (s, 2H), 1.42 (s, 9H), 1.27 (s, 9H), 1.24 (t, J = 7.0 Hz, 3H), 1.21 (t, J = 7.1 Hz, 3H).

**4-[(3,5-di-tert-butyl-2-ethoxyphenyl)(ethyl)amino]benzoic acid (MSU-41565).** A mixture of methyl 4-[(3,5-di-tert-butyl-2-ethoxyphenyl)(ethyl)amino]benzoate (0.064 g, 0.16 mmol), potassium hydroxide (0.087 g, 1.6 mmol), methanol (4.0 mL) and water (1.0 mL) was heated to 70^o^C overnight. The mixture was cooled and acidified with 1.0 N hydrochloric acid and extracted with ethyl acetate (3 times). The organic layers were combined, dried with sodium sulfate, filtered and the solvent removed in vacuo to provide a solid (0.034 g, 55% yield). 1H NMR (500 MHz, DMSO-d6) δ 12.17 (s, 1H), 7.71 (d, J = 8.8 Hz, 2H), 7.24 (d, J = 2.5 Hz, 1H), 6.95 (d, J = 2.4 Hz, 1H), 6.64 (d, J = 8.7 Hz, 2H), 3.84 (s, 2H), 3.62 (brm, 1H), 3.49 (brm, 1H), 1.37 (s, 9H), 1.23 (s, 9H), 1.16 (t, J = 7.0 Hz, 3H), 1.12 (t, J = 7.0 Hz, 3H). HRMS (ES-) (M-H) Calc. 396.2543, Found 396.2514.

**MSU-41846**

**5-bromo-1,3-di-tert-butyl-2-methoxybenzene.** 1,3-di-tert-butyl-2-methoxybenzene (Bai, Xinyan et al, Tetrahedron, 69(3), 1105-1111; 2013 (2.0 g, 9.1 mmol) was dissolved in acetonitrile (30.0 mL) and treated with n-bromosuccinimide (1.7 g, 9.5 mmol) and the mixture allowed to stir for 20 hours. The mixture was concentrated in vacuo then filtered through a SiO2 plug with hexanes. The solvent was then removed in vacuo to provide an oil (2.6g, 96%) used without further purification. 1H NMR (500 MHz, Chloroform-d) δ 7.34 (s, 2H), 3.68 (s, 3H), 1.41 (s, 18H).

**Methyl 4-[(3,5-di-tert-butyl-4-methoxyphenyl)amino]benzoate.** A mixture of 5-bromo-1,3-di-tert-butyl-2-methoxybenzene (0.30 g, 1.0 mmol), methyl 4-aminobenzoate (0.26 g, 1.7 mmol), cesium carbonate (0.65 g, 2.0 mmol), (±)-2,2′-Bis(diphenylphosphino)-1,1′-binaphthalene (0.062 g, 0.1 mmol) and palladium acetate (0.022 g, 0.10 mmol) in toluene (3.0 mL) was sealed in a microwave vial and heated by microwave to 110^o^C for 18 hrs. The mixture was then diluted into hexanes and Celite added and stirred. After 30 minutes, the mixture was filtered through a pad of Celite and the solvent removed in vacuo. The residue was purified via MPLC (SiO_2_, 100% hexanes gradient to 30% ethyl acetate) to provide a solid (0.21 g, 63%). 1H NMR (500 MHz, DMSO-d6) δ 8.59 (s, 1H), 7.80 – 7.73 (m, 2H), 7.04 (s, 2H), 6.97 – 6.90 (m, 2H), 3.75 (s, 3H), 3.63 (s, 3H), 1.37 (s, 18H).

**Methyl 4-[(3,5-di-tert-butyl-4-methoxyphenyl)(ethyl)amino]benzoate.** A solution of methyl 4-[(3,5-di-tert-butyl-4-methoxyphenyl)amino]benzoate (0.15 g, 0.41 mmol) in dimethylformamide (2.0 mL) at 0^o^C was treated with 60% sodium hydride (0.032 g, 0.81 mmol), stirred for 15 minutes, then treated with iodoethane (0.064 mL, 0.81 mmol) and the mixture stirred at room temperature for 4 hours. The mixture was then quenched with an aqueous solution of saturated ammonium chloride and extracted with ethyl acetate. The organic layers were combined, dried with sodium sulfate, filtered and the solvent removed in vacuo. The residue was then purified by MPLC (SiO_2_, 100% hexanes gradient to 10% ethyl acetate / hexanes) to provide a solid (0.164 g, quant.). 1H NMR (500 MHz, DMSO-d6) δ 7.75 – 7.67 (m, 2H), 7.04 (s, 2H), 6.64 – 6.59 (m, 2H), 3.74 (s, 3H), 3.73 – 3.69 (m, 2H), 3.67 (s, 3H), 1.36 (s, 18H), 1.13 (t, J = 7.0 Hz, 3H).

**4-[(3,5-di-tert-butyl-4-methoxyphenyl)(ethyl)amino]benzoic acid (MSU-41846).** A mixture of methyl 4-[(3,5-di-tert-butyl-4-methoxyphenyl)(ethyl)amino]-benzoate (0.16 g, 0.41 mmol), potassium hydroxide (0.23 g, 4.1 mmol), methanol (4.0 mL) and water (1.0 mL) was heated to 70^o^C for 2 days. The mixture was cooled and concentrated in vacuo. The residue was then redissolved in methanol and treated with 4.0 N HCl in dioxane (1.0 mL) and stirred for 20 minutes. The mixture was then filtered to remove solids and the solvent removed in vacuo to provide a solid (0.038, 25%). 1H NMR (500 MHz, Chloroform-d) δ 7.90 – 7.79 (m, 2H), 7.04 (s, 2H), 6.62 – 6.56 (m, 2H), 3.74 (s, 3H), 3.75-3.71 (m, 2H), 1.41 (s, 18H), 1.28 – 1.24 (m, 3H). HRMS (ES-) (M+H) Calc. 384.2523, Found 384.2572.

**References**

1 Wagner, C. E., Jurutka, P., Marshall, P. THERAPEUTIC COMPOUNDS. United States patent WO2016/140978 (2016).

2 Tashiro, M., Fukuda, Y. & Yamato, T. Selective preparation. 38. A convenient preparation of 2-(acylamino)biphenyls and N-acetylaniline derivatives using the tert-butyl group as a positional protective function. *The Journal of Organic Chemistry* **48**, 1927-1928, doi:10.1021/jo00159a036 (1983).
