# Supplementary material for: The novel rexinoid MSU-42011 is effective for the treatment of preclinical Kras-driven lung cancer
Source: Sci Rep. 2020 Dec 17;10:22244. doi: 10.1038/s41598-020-79260-8 (PMC7746742; doi:10.1038/s41598-020-79260-8)
Supplement: Supplementary file 2 — Supplementary Information [file 41598_2020_79260_MOESM2_ESM.pdf]

The novel rexinoid MSU-42011 is effective for the treatment of preclinical Kras-driven lung cancer

Jessica A. Moerland[1], Di Zhang[1], Lyndsey A. Reich[1], Sarah Carapellucci[1], Beth Lockwood[1], Ana S. Leal[1], Teresa Krieger-Burke[1,2], Bilal Aleiwi[1,3], Edmund Ellsworth[1,3], Karen T. Liby[1]

Authors' affiliations: [1]Department of Pharmacology & Toxicology, [2]In Vivo Facility, [3]Medicial Chemistry Core, Michigan State University, East Lansing, MI

Corresponding author: Karen T. Liby, Michigan State University, Department of Pharmacology and Toxicology, B430 Life Science Building, 1355 Bogue Street, East Lansing, MI 48824. Phone: 517-884-8955; Fax: 517-353-8915; Email: libykare@msu.edu

	SREBP mRNA fold expression change in HepG2 cells	Plasma triglyceride levels in mice (nmol/μL)
**Control**	1.0	1.08 ± 0.2
**Bexarotene**	2.23 ± 0.5	2.38 ± 0.3*
**LG100268**	3.06 ± 0.7	3.42 ± 0.4*
**LG101506**	1.06 ± 0.3	1.21 ± 0.1**

Supplemental Fig 1. Correlation of SREPB mRNA expression and triglyceride levels. HepG2 human liver cells were treated with 300 nM of rexinoids for 8 hr, and lysates analyzed by RT-PCR. Triglycerides in plasma from MMTV-neu mice fed rexinoids for 10 days were measured using a commercial kit (Abcam). *, $p < 0.01$ vs control; **, $p < 0.05$ vs bex and LG268; n=5-6 mice/group.

Supplemental Fig 2

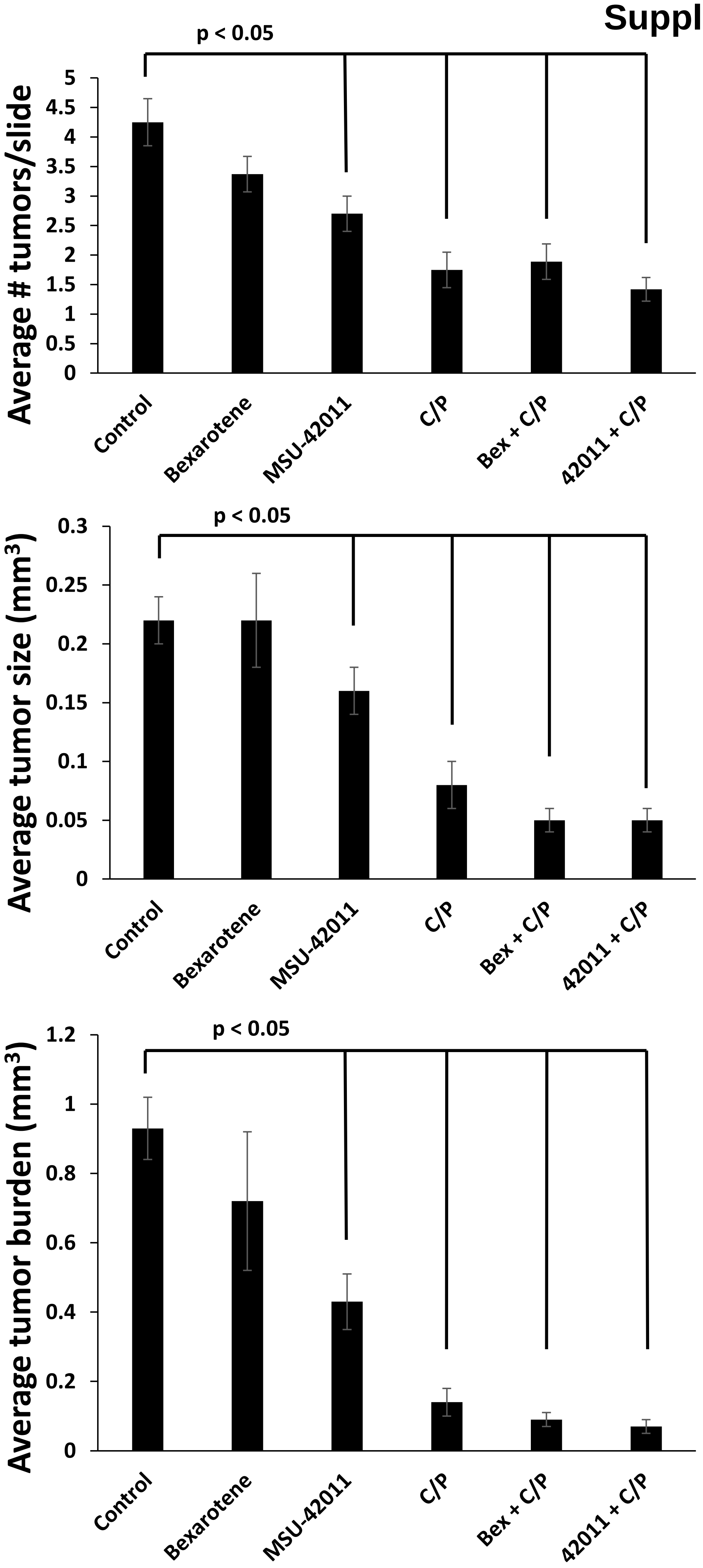

Supplemental Fig 2: MSU-42011 reduces tumor number, size, and burden. Female A/J mice were injected with 2 doses of vinyl carbamate (0.32 mg/injection), 1 week apart, to initiate lung carcinogenesis. After 8 weeks, mice were fed either control AIN-93G diet or rexinoids in the same diet (100 mg/kg diet or ~ 25 mg/kg body weight). One week after diets started, mice were injected i.p. every other week for a total of 6 injections with carboplatin (C - 50 mg/kg) and paclitaxel (P - 15 m/kg). After 12 weeks on diet, lungs were harvested and processed for sectioning and H & E staining. Values shown are mean ± SE.

Supplemental Fig 3

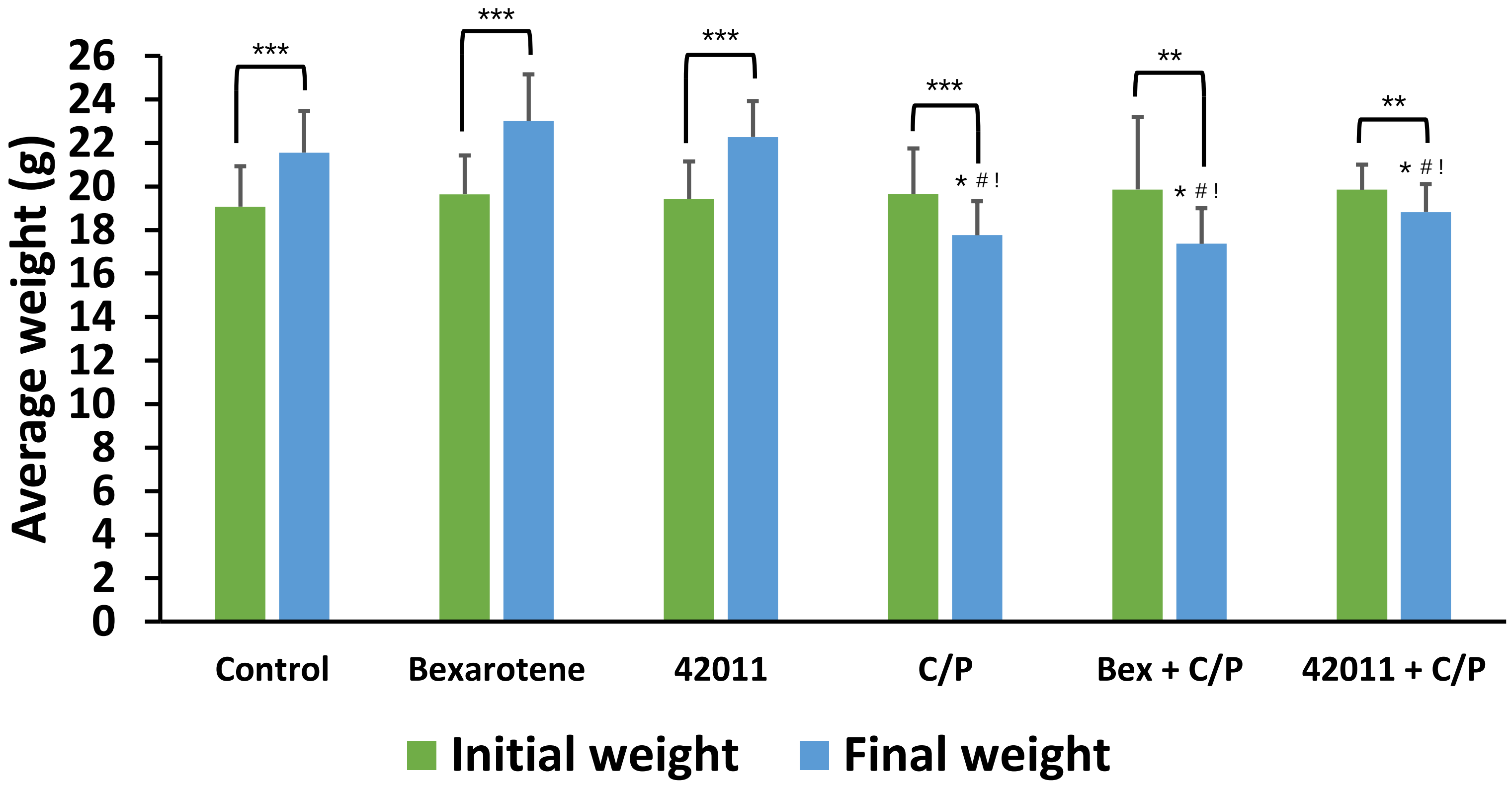

Supplemental Fig 3: Mouse weights. A/J mice were weighed weekly during the study. Values shown represent average mouse weights (n = 14-15/group) before treatment diets (initial weight at week 6) and at the end of the study (final weight at week 20). * $p < 0.01$ compared to control, # $p < 0.01$ compared to bexarotene, ! $p < 0.05$ compared to MSU-42011 (one-way ANOVA followed by Tukey HSD); ** $p < 0.05$, *** $p < 0.01$ (two-tailed t test).

Supplemental Fig 4

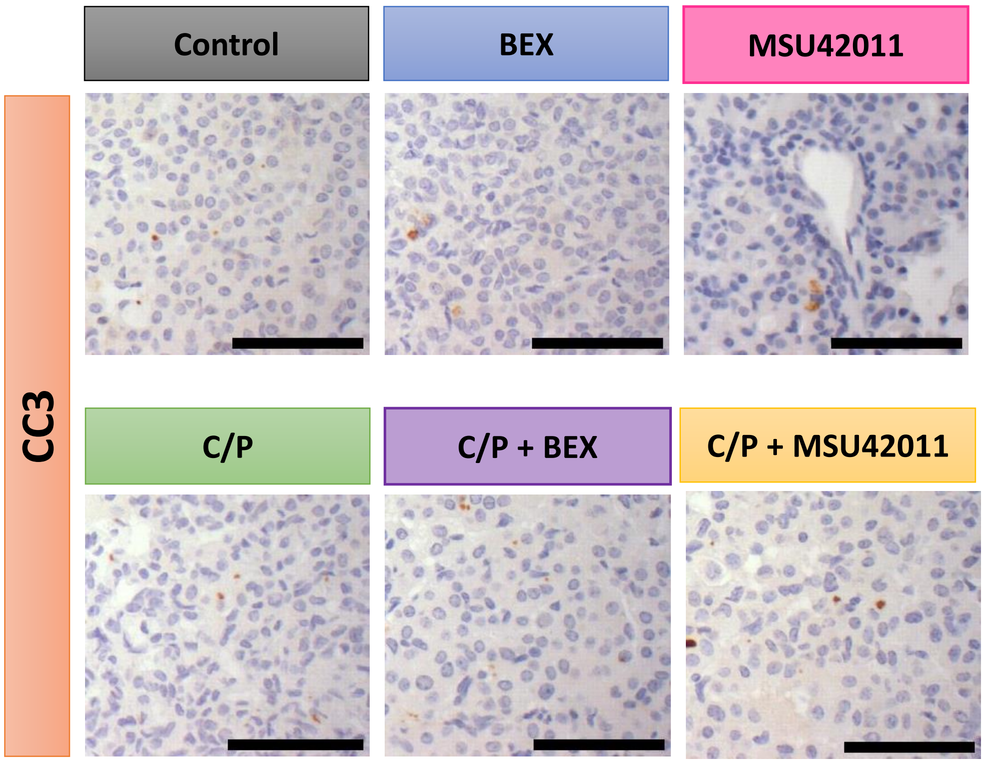

Supplemental Fig 4: Cleaved caspase 3 staining in lung tumors. A/J mice were injected with vinyl carbamate to induce lung tumors. After 8 weeks, mice were fed either control diet or rexinoids in the diet (100 mg/kg diet). One week after diets started, mice were injected i.p. with 6 total doses of carboplatin (C - 50 mg/kg) and paclitaxel (P - 15 m/kg), given every other week. After 12 weeks on diets, lungs were harvested and sectioned for immunohistochemical detection of cleaved caspase 3 (CC3). Scale bar represents 60 μM. BEX = bexarotene.

Supplemental Fig 5

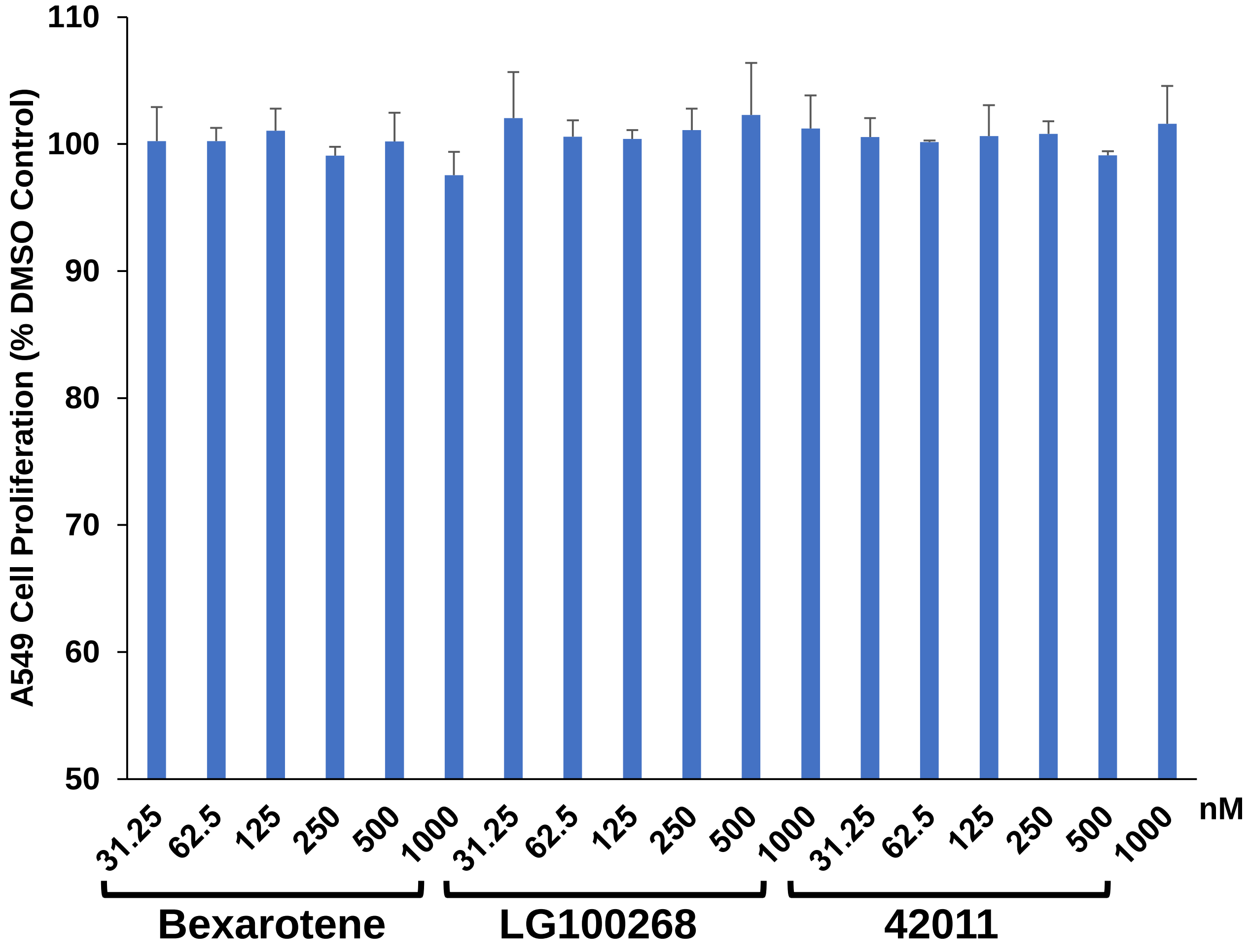

Supplemental Fig 5: Rexinoids do not inhibit proliferation of A549 lung cancer cells. A549 cells were treated with 0-1 μM rexinoids for 72 hr. Proliferation was measured using the MTT assay.

Supplemental methods

MTT Assay. A549 cells were plated in 96-well plates (2,000 cells/well). After overnight attachment, cells were treated with 2 fold dilutions (0-1000 nM rexinoids) for 72 hr. 50 μL 3-(4,5-dimethylthiazol-2-yl)-2,5-diphenyltetrazolium bromide (MTT; Sigma M2128) was added to each well, and cells were incubated at 37°C for 4 hr. Media was removed and cells were lysed with developing solution (0.04 M HCl in isopropanol). Plates were read at 550 nM.

Supplemental Fig 6

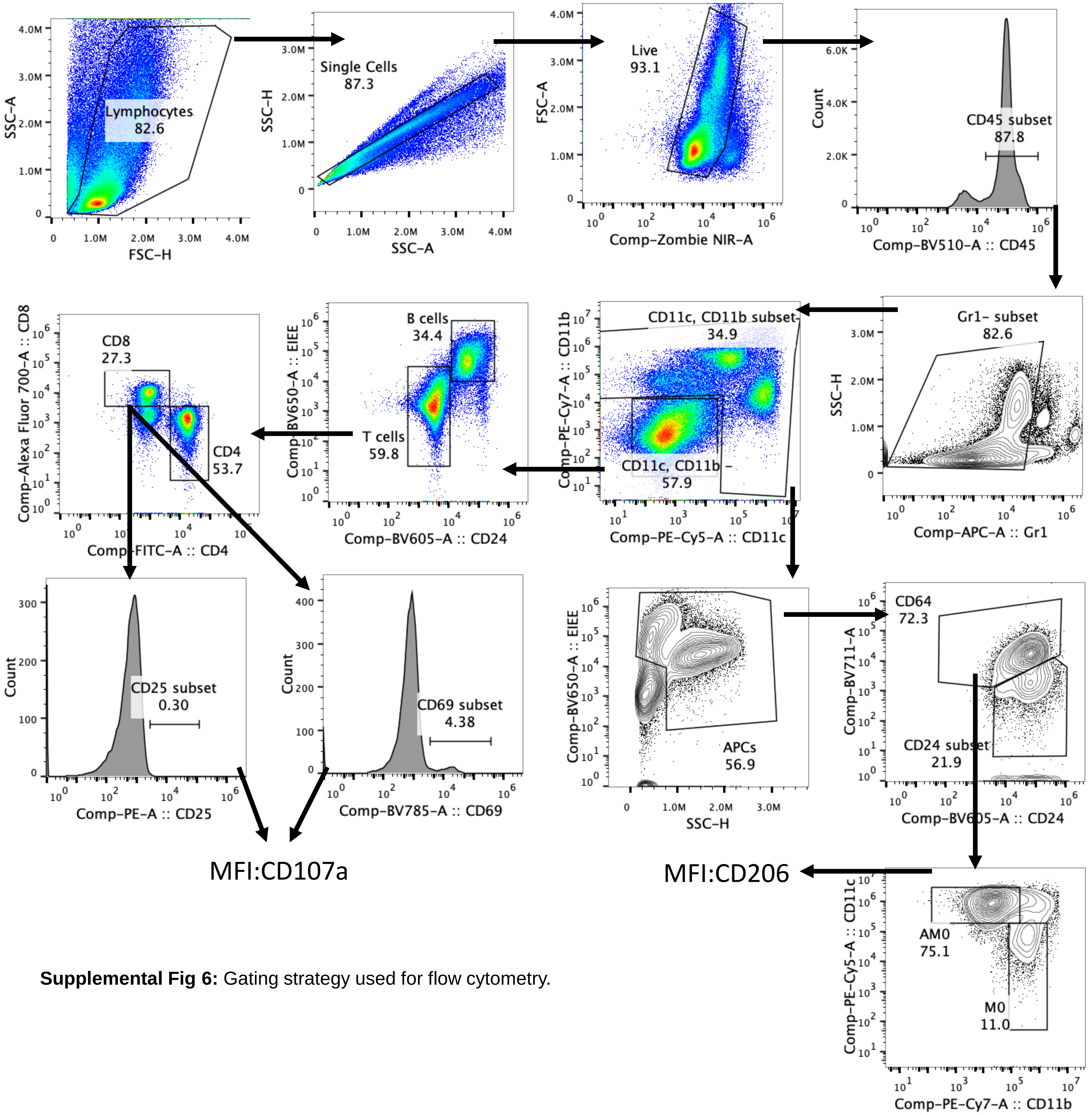

Supplemental Fig 6: Gating strategy used for flow cytometry.

Supplemental Fig 7

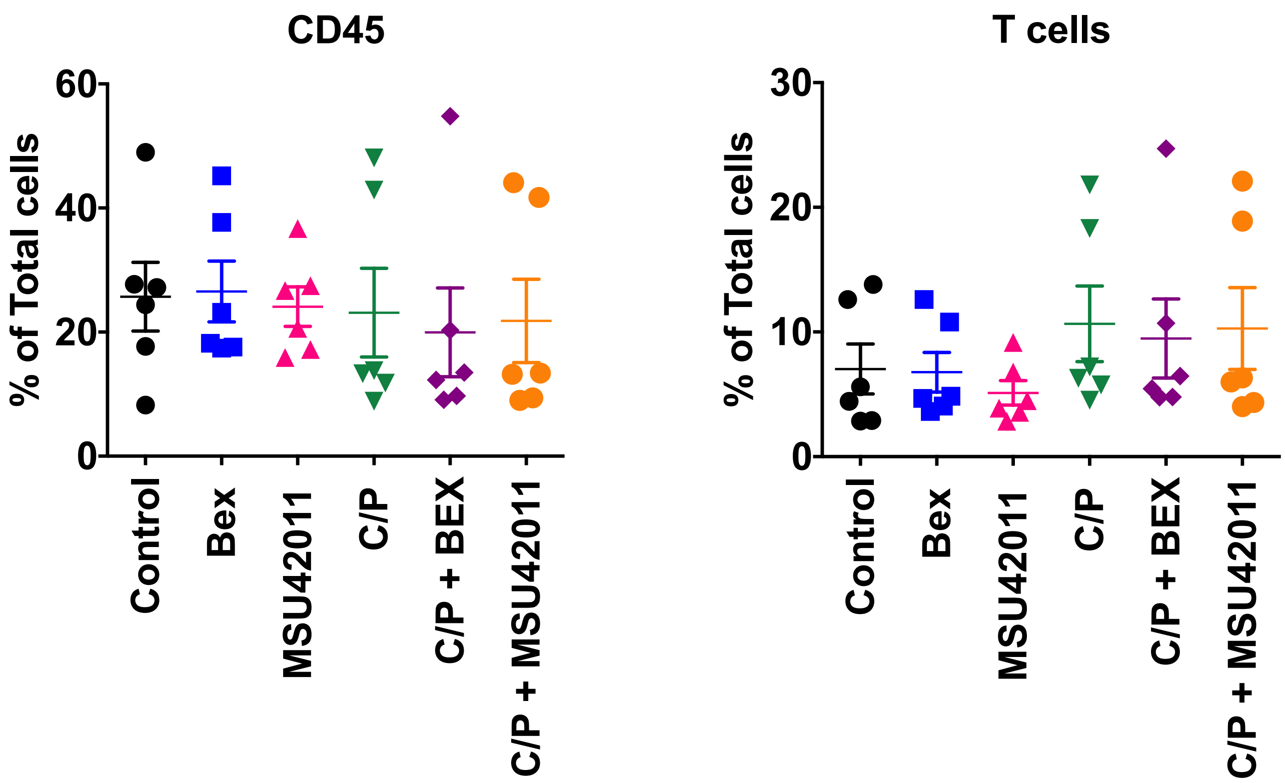

Supplemental Fig 7: Levels of total immune cells (CD45+) and CD3+ T cells in the lungs of A/J mice. Vinyl carbamate was used to initiate lung carcinogenesis. Eight weeks later, mice were fed experimental diets containing rexinoids (100 mg/kg) and treated with the combination of carboplatin and paclitaxel (C/P) as described in Table 1. After 12 weeks on diets, lung homogenates were incubated with appropriate antibodies and analyzed by flow cytometry. n = 6 mice/group.

Supplemental Fig 8

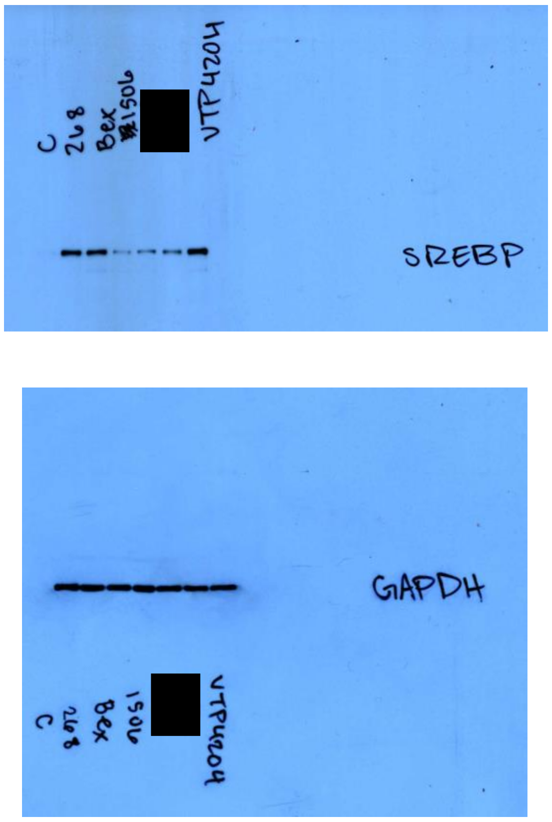

Immunoblots for Fig 1A

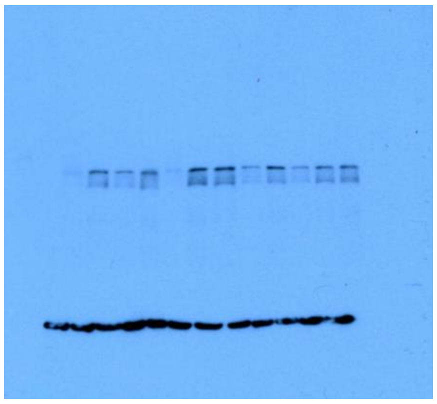

SREBP

GAPDH

Immunoblots for Fig 1D
